# Metabolic Heterogeneity in Patient Tumor-Derived Organoids by Primary Site and Drug Treatment

**DOI:** 10.3389/fonc.2020.00553

**Published:** 2020-05-15

**Authors:** Joe T. Sharick, Christine M. Walsh, Carley M. Sprackling, Cheri A. Pasch, Dan L. Pham, Karla Esbona, Alka Choudhary, Rebeca Garcia-Valera, Mark E. Burkard, Stephanie M. McGregor, Kristina A. Matkowskyj, Alexander A. Parikh, Ingrid M. Meszoely, Mark C. Kelley, Susan Tsai, Dustin A. Deming, Melissa C. Skala

**Affiliations:** ^1^Department of Biomedical Engineering, Vanderbilt University, Nashville, TN, United States; ^2^Morgridge Institute for Research, Madison, WI, United States; ^3^University of Wisconsin Carbone Cancer Center, Madison, WI, United States; ^4^Department of Biomedical Engineering, University of Wisconsin, Madison, WI, United States; ^5^Department of Pathology and Laboratory Medicine, University of Wisconsin, Madison, WI, United States; ^6^Department of Medicine, University of Wisconsin, Madison, WI, United States; ^7^Tecnológico de Monterrey, Escuela de Ingeniería y Ciencias, Zapopan, Mexico; ^8^William S. Middleton Memorial Veterans Hospital, Madison, WI, United States; ^9^Division of Surgical Oncology, East Carolina University Brody School of Medicine, Greenville, NC, United States; ^10^Department of Surgery, Vanderbilt University, Nashville, TN, United States; ^11^Department of Surgery, Medical College of Wisconsin, Milwaukee, WI, United States; ^12^Division of Hematology and Oncology, Department of Medicine, University of Wisconsin, Madison, WI, United States; ^13^McArdle Laboratory for Cancer Research, Department of Oncology, University of Wisconsin, Madison, WI, United States

**Keywords:** pancreatic cancer, breast cancer, organoid, optical metabolic imaging, heterogeneity, cellular metabolism

## Abstract

New tools are needed to match cancer patients with effective treatments. Patient-derived organoids offer a high-throughput platform to personalize treatments and discover novel therapies. Currently, methods to evaluate drug response in organoids are limited because they overlook cellular heterogeneity. In this study, non-invasive optical metabolic imaging (OMI) of cellular heterogeneity was characterized in breast cancer (BC) and pancreatic cancer (PC) patient-derived organoids. Baseline heterogeneity was analyzed for each patient, demonstrating that single-cell techniques, such as OMI, are required to capture the complete picture of heterogeneity present in a sample. Treatment-induced changes in heterogeneity were also analyzed, further demonstrating that these measurements greatly complement current techniques that only gauge average cellular response. Finally, OMI of cellular heterogeneity in organoids was evaluated as a predictor of clinical treatment response for the first time. Organoids were treated with the same drugs as the patient's prescribed regimen, and OMI measurements of heterogeneity were compared to patient outcome. OMI distinguished subpopulations of cells with divergent and dynamic responses to treatment in living organoids without the use of labels or dyes. OMI of organoids agreed with long-term therapeutic response in patients. With these capabilities, OMI could serve as a sensitive high-throughput tool to identify optimal therapies for individual patients, and to develop new effective therapies that address cellular heterogeneity in cancer.

## Introduction

Tumor organoids have emerged as an appealing method to tailor anti-cancer treatments by performing high-throughput drug screening directly on a patient's tumor cells (1–3). *In vitro* organoids, fully encapsulated in a basement membrane matrix, recapitulate the genetic and histopathological characteristics of the original tumor, along with its complex 3-dimensional organization (4–9). Organoid cultures also preserve interactions between tumor cells, immune cells (10), and fibroblasts (11), which can influence tumor drug response and are potential drug targets (12, 13). Generally, methods for measuring drug effects in organoids have involved either cell viability assays, pooling of proteins, DNA, and RNA from many organoids, or tracking of organoid diameter changes. These methods homogenize the response of an entire organoid or many organoids and ignore cellular heterogeneity, which drives tumor treatment resistance (14–17). It is possible for minority subpopulations of lethal drug-resistant cells to go completely undetected without more advanced assessment tools. Additionally, these methods generally neglect cellular metabolism, which is a major factor determining cellular drug response and heterogeneity (18–20). A study of inter-tumor metabolic heterogeneity detected unique metabolomic profiles in each of over 180 melanoma patient tumors (21), highlighting the importance of metabolism in personalized medicine.

Optical metabolic imaging (OMI) is a novel, non-destructive, high-resolution fluorescence microscopy technique that quantifies the metabolic state of individual cells within a single organoid using cellular autofluorescence (22, 23). The fluorescence properties of NADH and NADPH overlap and are referred to as NAD(P)H. NAD(P)H, an electron donor, and FAD, an electron acceptor, are fluorescent metabolic co-enzymes present in all living cells. The optical redox ratio, defined as the ratio of the fluorescence intensity of NAD(P)H to that of FAD, reflects the redox state of the cell (24–26), and is sensitive to shifts in metabolic pathways (23, 27, 28). The fluorescence lifetimes of NAD(P)H and FAD are both two-exponential with distinct lifetimes for the free- and protein-bound conformations, and thus reflect the protein-binding activities of NAD(P)H and FAD (29–31). The lifetime of free NAD(P)H is shorter than bound NAD(P)H, and conversely, free FAD is longer than bound FAD. As a result, fluorescence lifetime imaging microscopy (FLIM) of endogenous biomarkers detects early metabolic changes in response to anti-cancer drug treatment (32–34). The optical redox ratio, NAD(P)H, and FAD fluorescence lifetimes all provide complementary information, and can be combined into a composite endpoint called the OMI index (35). This metric distinguishes drug-resistant and responsive cells by their metabolic states and is robust and sensitive in pancreatic and breast cancer organoids (1, 35).

OMI of organoids could improve predictions of patient outcomes for several reasons. First, drug-induced changes in cell metabolism measured by OMI precede changes in tumor size or overall cell viability (1, 23, 35, 36), and thus can measure drug response faster than conventional methods such as apoptosis and proliferation assays. Second, OMI analysis of cell subpopulations identifies and quantifies tumor heterogeneity (36, 37), which is vital for accurately capturing patient drug response. Finally, OMI is non-invasive and does not require exogenous labels, so treatment response can be tracked over time in the same organoids. This is not possible with standard techniques which, by necessity, destroy samples. Therefore, OMI could provide a fast, dynamic method to evaluate heterogeneous drug response at the organoid and single-cell level, and therefore integrate tumor heterogeneity into clinical treatment planning and pre-clinical drug discovery.

In this study, cellular metabolic heterogeneity in patient organoids is characterized using a panel of quantitative techniques for the first time. Intra-tumor heterogeneity at baseline is compared across OMI variables and tumor types, and intra-organoid heterogeneity at baseline is compared between organoid morphology types and tumor types. OMI of organoids has been validated as an accurate predictor of *in vivo* drug response in mouse models of pancreatic cancer (PC) (1), xenografts generated from human breast cancer (BC) cell lines (35), and a colorectal cancer patient (38), but has not yet been evaluated for primary human pancreatic and breast tumors. Currently, oncologists must weigh drug treatment options for individual PC patients based solely on potential side effects and have no *a priori* indication of whether a PC patient will respond to standard therapies. Clinicians select treatments for BC patients based on pathological analysis of hormone receptors and human epidermal growth factor receptor 2 (HER2). Response to treatment in both PC and BC is defined by tracking tumor size with imaging, but evidence of treatment failure can require months of observation. Tailoring treatment based on genomic analysis alone has proven insufficient due to poor understanding of the connections between tumor driver mutations and drug response (39). Recurrences in both BC and PC could be minimized with technologies to quickly and accurately determine an optimal treatment plan. This study is the first to demonstrate that OMI of cellular metabolic heterogeneity in pancreatic and breast tumor organoids could provide an early measure of long-term *in vivo* drug response for individual patients.

## Materials and Methods

### PC Tissue Processing and Organoid Culture

Human tissue was collected with informed consent from all patients, and all studies were approved by the Institutional Review Boards at the University of Wisconsin-Madison and the Medical College of Wisconsin (IRB# 2018-1104). Surgically resected tissue was placed in cold chelation buffer on ice for 1 h. Tissues were washed with phosphate buffered saline (PBS) and digested at 37°C in Dulbecco's Modified Eagle's Medium: Nutrient Mixture F-12 (DMEM/F-12) medium (Invitrogen) containing 1 mg/mL collagenase (Sigma), 0.125 mg/mL dispase (Invitrogen), 10% fetal bovine serum (FBS) (Gibco), and 1% pen-strep (Gibco) for 2–3 h with intermittent shaking. The resulting cell macro-suspension was rinsed in cold PBS, re-suspended in a 1:1 mixture of DMEM/F-12 media and Matrigel extracellular matrix (Corning), plated in 50 μl droplets, and allowed to solidify at 37°C, 5% CO_2_ in a cell incubator. Once solidified, droplets were overlaid with DMEM/F-12 supplemented with 7% FBS, 20 μM Y-27632 (Sigma), 50 ng/ml epidermal growth factor (EGF) (Invitrogen), R-Spondin (RSPO)-conditioned medium (homemade) and 1% penicillin-streptomycin (Gibco). FBS, Y-27632, and RSPO-conditioned medium were removed from cultures if fibroblasts were out-growing tumor cells.

### BC Tissue Processing and Organoid Culture

Human tissue was collected with informed consent from all patients in accordance with HIPAA regulation, and all studies were approved by the Institutional Review Board at the University of Wisconsin-Madison (IRB# UW14035). Patients with very high risk of having breast cancer were identified as those with breast imaging reporting and data system (BI-RADS) scores of 4C, 5, or 6 that had not received prior treatment. For these patients, an additional tumor sample was obtained during a diagnostic biopsy and immediately placed in sterile PBS. Tissue was enzymatically digested in BC organoid media (9) containing 1.5 mg/mL collagenase for 45–90 min at 37°C, with intermittent shaking. The resulting cell suspension was rinsed in cold PBS and resuspended in cold BC organoid media. All cellular solutions were mixed at a 1:1 ratio with Matrigel and deposited as 50 μl droplets into 35 mm glass-bottom dishes. Gels were allowed to solidify at 37°C, 5% CO_2_ in a cell incubator for 15–30 min, and BC organoid media (9) was added.

### Drug Screening

Twenty-four hours prior to imaging, media was replaced with fresh media containing drugs, including 85 μM gemcitabine (40–42), 10 μM 5-FU (38, 43), 200 nM TAK-228 (44, 45), 250 nM ABT-263 (46, 47), 5 μM oxaliplatin (38, 48), 10 μM nab-paclitaxel (41), 50 nM SN-38 (49), 500 nM paclitaxel (41), 2 μM docetaxel (50), 1.5 μM 4-OOH cyclophosphamide (51), 5 μM doxorubicin (52), 10 μg/mL trastuzumab (53), 25 μg/mL pertuzumab (54), or combinations of each. Doses were selected to replicate clinically relevant peak plasma concentrations. FOLFIRINOX treatment was comprised of 5-FU, oxaliplatin, and SN-38. Not all drugs were tested on every patient's cells for the full time-course. Drug choices were made based on the availability of viable organoids and the clinical treatment plans (or lack thereof) for individual patients. All organoids were initially treated with a standard panel because gemcitabine and 5-FU combination therapy was most likely to be prescribed after surgery (excluding Patient PC6; it was known in advance that they would receive oxaliplatin rather than gemcitabine). When additional drugs were prescribed for a patient such as nab-paclitaxel or the FOLFIRINOX regimen, their panels were expanded to incorporate those treatments. After the first imaging time point (24 h), gemcitabine, nab-paclitaxel, SN-38, and oxaliplatin were removed from cultures to simulate the delivery of one dose, while exposure of all other drugs was maintained throughout the experiment to simulate daily delivery. Chemotherapy drugs were obtained from the University of Wisconsin Carbone Cancer Center Pharmacy. TAK-228 was obtained from LC Laboratories, ABT-263 was obtained from Apex Bio, and 4-OOH cyclophosphamide was obtained from Santa Cruz Biotech.

### Multiphoton Imaging

Fluorescence imaging was performed using a custom multiphoton fluorescence lifetime system (Bruker Fluorescence Microscopy). A 40x water immersion objective [Nikon, 1.15 numerical aperture (NA)] was used with an inverted microscope (Nikon, TiE). A titanium:sapphire laser (Spectra-Physics InSight DS+) was used for excitation, while gallium arsenide phosphide (GaAsP) photomultiplier tubes (H7422P-40, Hamamatsu) detected emission light. 750 and 890 nm light were used for two-photon excitation of NAD(P)H and FAD, respectively. A 440/80 nm filter was used to collect NAD(P)H fluorescence emission, and a 550/100 nm filter was used to collect FAD fluorescence emission. Images were acquired over 60 s, with a pixel dwell time of 4.8 μs for 256 × 256 pixel images. Fluorescence lifetime data with 256 time bins was acquired using time-correlated single photon counting electronics (SPC-150, Becker & Hickl). A Fluoresbrite Yellow Green microsphere (Polysciences) was imaged daily as a fluorescence lifetime standard, which had a stable lifetime (2.07 ± 0.05 ns, *n* = 86), consistent with previously published values (22, 29, 31).

### Organoid Imaging

Imaging of organoids was performed in 35 mm glass-bottom dishes (#P35G-1.5-14-C, MatTek). At least five representative organoids were imaged near the center of the organoid in each treatment group at each time point. At least two additional images of the fibroblast monolayer on the coverslip were taken, if present. Images were acquired 1, 2, and 3 days after initial treatment, and at day 5 and day 7 in some cases. For two patients, drug treatment experiments were not performed due to low organoid count, and only baseline heterogeneity was imaged (BC21 + BC22).

### Image Analysis

NAD(P)H and FAD fluorescence lifetime images were analyzed using SPCImage software (Becker & Hickl) (55). Briefly, a histogram of photon counts per temporal bin, or decay curve, is generated for each pixel by binning the photon counts of all 8 surrounding pixels. This decay curve is deconvolved with the instrument response function, and then fit to a two-component exponential decay (Equation 1).

(1) $$\begin{array}{l} I\left(t\right)=\alpha_{1}exp^{-t/\tau_{1}}+\;\alpha_{2}exp^{-t/\tau_{2}}+\;C \end{array}$$

Here, *I*(*t*) represents the fluorescence intensity measured at time *t*, α_1_, and α_2_ represent the fractional contributions of the short and long lifetime components to the overall signal, respectively, τ_1_ and τ_2_ are the short and long lifetime components, respectively, and *C* represents background light. The two lifetime components are used to distinguish between the free and bound forms of NAD(P)H and FAD (56, 57). The mean lifetime (τ_*m*_) is a weighted average of the free and bound lifetimes, and is calculated for both NAD(P)H and FAD in each pixel using Equation 2.

(2) $$\begin{array}{l} \tau_{m}=\alpha_{1}\;*\;\tau_{1}+\alpha_{2}\;*\;\tau_{2} \end{array}$$

The decay curves for NAD(P)H and FAD were integrated for each pixel to obtain intensity values. The optical redox ratio was calculated for each pixel by dividing the intensity of NAD(P)H by the intensity of FAD. A customized CellProfiler routine was written to automatically identify individual cell cytoplasms and extract average NAD(P)H and FAD intensities and lifetime components for each (58, 59). All reported redox ratios are normalized to average control values of the same patient and time point.

### OMI Index Calculation

The OMI index, in this study, is a linear combination of three independent OMI endpoints [redox ratio, NAD(P)H τ_*m*_, and FAD τ_*m*_], each centered around the average value measured in control cells within each patient at the same experimental time point. This differs from previous descriptions (1, 35) where the end points are mean-centered across all cells in all treatment groups. This modification allows drug responses to be compared between patients in this study. As before, the redox ratio, NAD(P)H τ_*m*_, and FAD τ_*m*_ are given coefficients of (1,1,−1). A decrease in OMI index relative to control correlates with drug response, while an increase or lack of change indicates drug resistance.

### Organoid Heterogeneity Analysis

A Gaussian mixture distribution model was used to assess heterogeneity of cellular metabolism (1, 35, 37, 60, 61). OMI values for all cells within a treatment group and time point are inputted into this model described by Equation (3).

(3) $$\begin{array}{l} f\left(y;\Phi_{g}\right)=\overset{g}{\underset{i=1}{\sum }}\pi_{i}\phi \left(y;\mu_{i},V_{i}\right) \end{array}$$

Here, *g* represents the number of subpopulations in the model, ϕ(*y*; μ_*i*_, *V*_*i*_) is a normal probability density function where μ_*i*_ represents the mean and *V*_*i*_ represents variance, and π_*i*_ is the mixing proportion. Models containing 1, 2, and 3 subpopulations are fit to the data, and the goodness of fit for each model is assessed using the Akaike information criterion (AIC) (62). The best fit of the three models, equivalent to the lowest AIC, is used to evaluate heterogeneity. For comparison, distributions are normalized such that all have an area of 1 under the curve. We previously defined and validated the weighted heterogeneity index (wH-index, Equation 4) to predict *in vivo* treatment response with OMI in mouse models of breast cancer (61). The wH-index is based on the Gaussian distribution models described by Equation (3), and is a modified form of the Shannon diversity index used to quantify the degree of heterogeneity in a population (36, 63).

(4) $$wH-index=\sum \left(1-p_{i}ln\left(p_{i}+1\right)\right)*\left(\sigma_{i}+d_{i}\right)$$

Here, *i* represents each subpopulation in the Gaussian distribution model, *d* represents the distance between the median of each subpopulation and the median of the entire distribution, *p* represents the proportion of all cells belonging to that subpopulation, and σ is the standard deviation. Quadratic entropy (QE), Kolmogorov-Smirnov distance (KS), and outlier percentage (OL) were calculated for each treatment group's cellular distribution as described previously (64).

### Patient Tumor Heterogeneity Analysis

Standard hematoxylin and eosin-stained sections from breast tissue collected in parallel with the harvested tissue were assessed histologically for cytologic variability based on nuclear size and chromatin appearance by a board-certified pathologist with sub-specialty training in breast pathology (SMM). Cases in which all nuclei appeared similar were regarded as having low cytologic variability and cases that had marked variation in nuclear appearance from low magnification were regarded as having high variability; cases with variation that could only be appreciated at higher magnification were classified as moderate.

For pancreatic cancer analysis, hematoxylin and eosin-stained sections from samples acquired at the time of resection and submitted for research use were reviewed. In addition, hematoxylin, and eosin-stained sections from the original diagnostic resection specimen were reviewed by a board-certified pathologist with sub-specialty training in gastrointestinal and pancreaticobiliary pathology (KAM). All slides were assessed histologically for tumor heterogeneity based on tumor differentiation, which incorporates growth pattern and presence/absence of gland formation. Cases with abundant gland formation were regarded as well-differentiated tumors, while those with minimal gland formation, contained single infiltrating cells, or a solid growth pattern were regarded as poorly differentiated. Tumors with similar histologic findings across the sampled tumor were consistent with low tumor heterogeneity/variability, while cases with differences in tumor growth pattern and morphology were regarded as having more tumor heterogeneity/variability.

### Cyanide Experiment

Four previously untreated organoids from Patient PC1 underwent OMI immediately before and after the addition of media containing NaCN (Sigma), for a final concentration of 6 mM. OMI endpoints were quantified at the single-cell level, at least 60 cells per group. Redox ratio values were normalized to the pre-treatment average.

### Organoid Immunofluorescence

Organoids were rinsed in PBS and fixed for 20 min in 4% paraformaldehyde (VWR). Fixed organoids were rinsed and stored in PBS at 4°C until stained. Organoids were blocked for 1 h in PBS with 10% goat serum and 0.3% Triton-X 100 (Sigma) at room temperature followed by co-incubation with Ki67 antibody conjugated to AlexaFluor 488 (1:50, Cell Signaling #11882S) and CC3 antibody conjugated to AlexaFluor 555 (1:50, Cell Signaling #9604S) for 48 h at 4°C. Organoids were then washed in PBS and mounted to a slide using ProLong™ Diamond Antifade Mountant with DAPI (Molecular Probes). DAPI was imaged on the multiphoton microscope at 40x magnification using 750 nm for excitation and 440/80 nm bandpass filter for emission. AlexaFluor 488 was imaged using 965 nm for excitation and 550/100 nm filter for emission. AlexaFluor 555 was imaged using 1,050 nm excitation and a 585/65 nm filter. Six or more organoids per treatment group were imaged and the percentage of Ki67-positive cells and cleaved caspase-3 (CC3) positive cells in each organoid were quantified.

### PC Patient-Derived Xenografts

Animal research was approved by the UW-Madison Institutional Animal Care and Use Committee. Organoids from Patient PC13 were pelleted, re-suspended in media, and mixed 1:1 with Matrigel. This mixture was subsequently injected (100 μl) subcutaneously into bilateral flanks of female NOD *scid* gamma mice at 6 weeks old (NOD.Cg-*Prkdc*^*scid*^ *Il2rg*^*tm*1*Wj*1^/SzJ, The Jackson Laboratory) for initial patient-derived xenograft (PDX) establishment. For treatment experiments, extracted tumors were mechanically minced to form a cell suspension, which was then mixed with Matrigel for injection into experimentally naïve female athymic nude mice at 6 weeks old (Hsd:Athymic nude-Foxn1^nu/nu^, Envigo). Tumor volume was measured with calipers using the formula 0.5 * length * width^2^. When average tumor volume reached ~150 mm^3^, mice were randomized into two groups. Twenty mice received 100 mg/kg gemcitabine and 100 mg/kg nab-paclitaxel weekly via intraperitoneal injection while 23 control mice received only PBS weekly. Tumor volume was measured twice weekly. Mice were euthanized and tumors were collected when humane endpoints were reached.

### High-Depth Targeted Gene Sequencing

Patient PC13 organoids were sequenced using the Qiagen Comprehensive Cancer Panel and molecular barcode technology, with >500x median coverage.

### Statistics

Differences in OMI index, redox ratio, NAD(P)H τ_*m*_, FAD τ_*m*_, CC3+%, Ki67+%, standard deviation, coefficient of variation (CV), QE, and KS between groups were tested using a Wilcoxon rank-sum test. This test was chosen because these data distributions were not assumed to be parametric in nature. Normalized tumor volumes were compared using a student *t*-test and a D'agostino-Pearson normality test. Treatment effect size was calculated with Glass's Δ (65) because comparisons of very large sample sizes of individual cells always pass traditional significance tests unless the population effect size is truly zero. Linear regression modeling was performed using ordinary least squares fitting, and the adjusted coefficient of determination was used to report the percentage of the variance in the dependent variable that can be explained by the variance of the independent variables in all linear models.

## Results

### Patient-Derived Tumor Organoid Generation and Optical Metabolic Imaging

**Figure 1:**
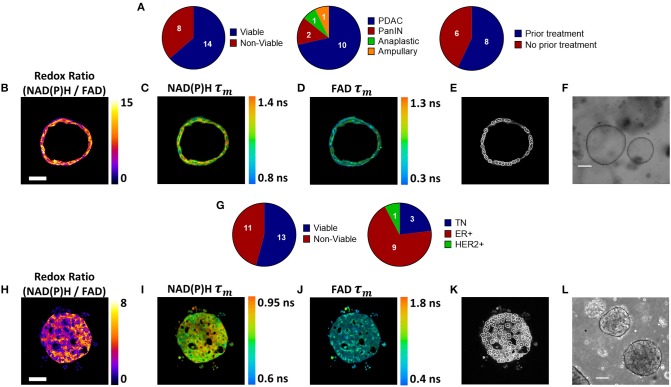
Patient-derived tumor organoid generation and optical metabolic imaging. **(A)** Pie charts depicting the success rate for generating viable organoids from patient pancreatic lesions (left), the distribution of PDAC, PanIN, anaplastic cancer, and ampullary cancer among successfully generated organoids (center), and the distribution of previously treated vs. untreated tumors among successfully generated organoids (right). Representative redox ratio **(B)**, NAD(P)H τ_*m*_ **(C)**, and FAD τ_*m*_ **(D)** images of an untreated pancreatic organoid taken 6 days after surgical resection (Patient PC14). Scale bar is 50 μm **(E)**. Masks of individual cell cytoplasms overlaid onto NAD(P)H intensity image **(F)**. Representative brightfield image of pancreatic organoids (Patient PC14). Scale bar is 200 μm. **(G)** Pie charts depicting the success rate for generating viable organoids from patient breast tumor biopsies (left), and the distribution of receptor subtypes among successfully generated organoids (right). Representative redox ratio **(H)**, NAD(P)H τ_*m*_ **(I)**, and FAD τ_*m*_ **(J)** images of an untreated breast cancer organoid taken 30 days after biopsy (Patient BC9). Scale bar is 50 μm. **(K)** Masks of individual cell cytoplasms overlaid onto NAD(P)H intensity image. **(L)** Representative brightfield image of breast cancer organoids (Patient BC9). Scale bar is 200 μm.

Pancreatic organoids were generated from fresh patient tissue samples acquired during distal pancreatectomy or pancreaticoduodenectomy (Whipple resection) surgeries. The overall rate of successful organoid formation was 64% (14 of 22 patients) (Figure 1A), including mostly pancreatic tumors (pancreatic ductal adenocarcinomas (PDAC) and anaplastic carcinoma of the pancreas), along with two pancreatic intraepithelial neoplasia (PanIN) lesions and one ampullary adenocarcinoma (Supplementary Table S1). Fifty-seven percent of the successfully cultured patient samples underwent neoadjuvant treatment prior to resection, including one patient that was downgraded from PDAC to PanIN following a complete pathologic response to chemotherapy (Patient PC5). Neoadjuvant treatment did not impede organoid formation (67 and 60% success rates for pretreated and non-pretreated samples, respectively). Representative images demonstrate that multiphoton microscopy measures OMI endpoints with high resolution (Figures 1B–D). This allows endpoints to be quantified in individual cells by masking each cell nucleus and cytoplasm using NAD(P)H fluorescence intensity (Figure 1E). Tumor cells grew as both 3-dimensional hollow spheres (Figure 1F) as well as solid spheres. The length of the organoid establishment period varied by PC patient between 4 and 33 days (Supplementary Table S1), and ended when organoids were clearly visible and proliferating with rounded edges. Only two organoid lines required more than 12 days of establishment time. Differences in size and cellular quality of patient tumor samples likely contributed to the variance in time to maturation for organoid lines.

BC organoids were generated from core needle biopsies of suspected tumors, acquired at the time of initial diagnosis. Organoids were successfully generated in 54% of cases (13 of 24), including patients with a variety of receptor statuses (Figure 1G, Supplementary Table S2). Again, OMI endpoints were acquired with high resolution (Figures 1H–J), and NAD(P)H fluorescence intensity images were used to mask each cell cytoplasm (Figure 1K). Similar to pancreas organoids, both hollow and solid (Figure 1L) morphologies were noted in BC organoids.

### Sources of Baseline Metabolic Variability in Patient-Derived Organoid Cells

**Figure 2:**
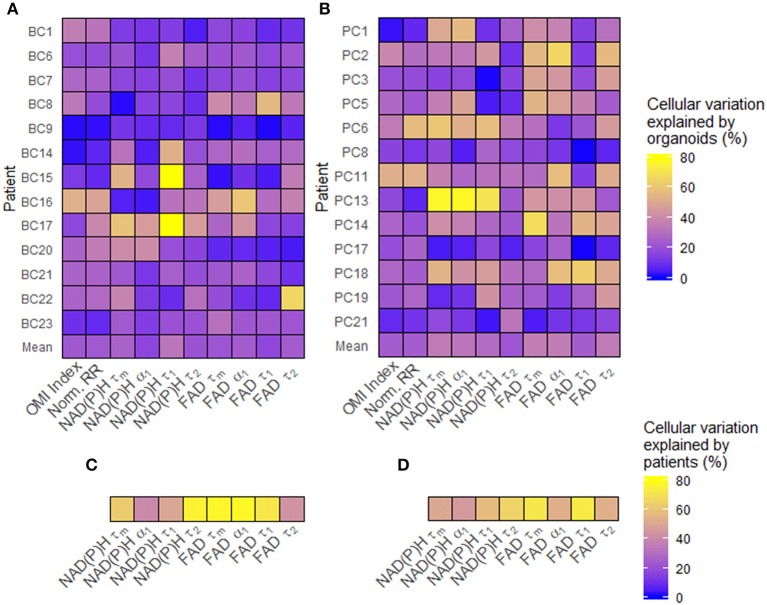
Sources of baseline metabolic variability in patient-derived organoid cells. **(A)** The percentage of total metabolic variation among each breast cancer patient's cells which can be explained by variation at the organoid level for each variable. **(B)** The percentage of total metabolic variation among each pancreatic patient's cells which can be explained by variation at the organoid level for each variable. **(C)** The percentage of total metabolic variation among cells from all breast cancer patients which can be explained by variation between patients for each variable. **(D)** The percentage of total metabolic variation among cells from all pancreatic patients which can be explained by variation between patients for each variable. Untreated organoid cells from the first measurement time point were included in this analysis.

Single-cell data from untreated organoids was used to characterize baseline cellular metabolic heterogeneity and its origin. The percentage of variation between individual cells in untreated organoids that are due to differences at the organoid level were quantified using linear modeling for each patient and OMI variable (Figures 2A,B). Higher percentages suggest that a larger portion of overall cellular metabolic heterogeneity within a patient is due to inter-organoid variability, while lower percentages suggest that a larger portion is due to intra-organoid variability. This percentage estimates the likelihood of accurately predicting the metabolic properties of a single cell based solely on the properties of its individual organoid of origin. When averaged across all patients of a cancer type, averages for each of the 10 variables ranged between 16 and 36%. Next, the fraction of overall cellular metabolic variation that was due to differences at the patient level were quantified for each variable (Figures 2C,D), ranging from 39 to 78%. In all cases, the proportion of cellular variation due to patient-to-patient variation (Figures 2C,D) was higher than the mean proportion of cellular variation due to organoid-to-organoid variation (Figures 2A,B). Additional established indices for quantifying cellular heterogeneity, including wH-index (uses Gaussian fitting to identify subpopulations) (61), KS (a measure of distribution normality) (64), QE (a quantitative measure of the number of species in a population) (64), and OL (cells that are distinct from the majority) (64) were applied at the patient level to all baseline organoids. Bivariate correlations between all heterogeneity and mean fluorescence lifetime measurements, as well as patient age at the time of tissue acquisition, were performed (Supplementary Figure S1). Many measurements exhibit few or zero correlations with any others, suggesting that they provide unique information about the metabolism or metabolic heterogeneity of the patient's organoid cells. An orthogonal measurement of intra-tumor heterogeneity defined by a pathologist (SMM), cytologic variability, was compared to corresponding OMI measurements of baseline patient heterogeneity in organoids (Supplementary Figure S2). Redox ratio KS and FAD τ_*m*_ QE were significantly higher in BC patients with medium/high cytologic variability compared to patients with low cytologic variability. Cytologic variability categories in PC (defined by a pathologist, KAM) did not have significantly different baseline OMI heterogeneity for any variable (*p* > 0.05).

### Disparities in Metabolism and Intra-Organoid Heterogeneity by Morphology and Cancer Type

**Figure 3:**
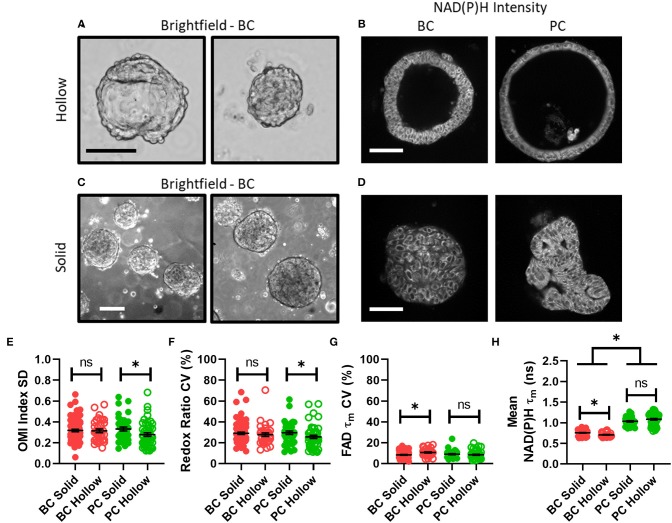
Disparities in metabolism and intra-organoid heterogeneity by morphology and cancer type. **(A)** Representative brightfield images of hollow breast cancer organoids. Scale bar = 100 μm. **(B)** Representative NAD(P)H intensity images of hollow breast and pancreatic cancer organoids. Scale bar = 100 μm. **(C)** Representative brightfield images of solid breast cancer organoids. Scale bar = 250 μm. **(D)** Representative NAD(P)H intensity images of solid breast and pancreatic cancer organoids. Scale bar = 100 μm. **(E–H)** The intra-organoid standard deviation of the OMI index **(E)**, coefficient of variation of the redox ratio **(F)**, coefficient of variation of mean fluorescence lifetime of FAD **(G)**, and the mean fluorescence lifetime of NAD(P)H **(H)** for cells within hollow vs. solid organoids. *p < 0.05. Each dot represents one organoid (mean ± SEM).

Next, organoid morphology was evaluated as a potential factor in intra-organoid metabolic heterogeneity. All untreated organoids at initial imaging time points were classified as either morphologically hollow (Figures 3A,B) or solid (Figures 3C,D) based on images of NAD(P)H intensity. In PC, hollow organoids exhibited significantly less metabolic heterogeneity than solid organoids in terms of OMI index SD (Figure 3E) and redox ratio CV (Figure 3F). In BC, hollow organoids exhibited significantly more metabolic heterogeneity than solid organoids in terms of FAD τ_*m*_ CV (Figure 3G), and significantly shorter mean NAD(P)H τ_*m*_ values (Figure 3H). Other comparisons were not significantly different between hollow and solid organoids (Supplementary Figure S3).

### OMI of PC Organoids Resolves Differential Sensitivities to Relevant Drug Treatments

**Figure 4:**
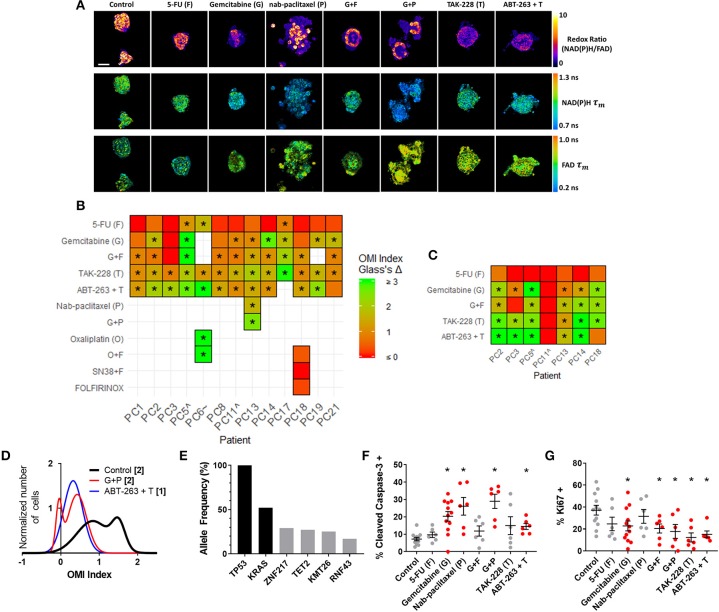
OMI captures non-genetic cellular heterogeneity in pancreatic organoids. **(A)** Representative images of the redox ratio, NAD(P)H τ_m_, and FAD τ_m_, in organoids generated from Patient PC13 (anaplastic carcinoma of the pancreas), treated with standard chemotherapies and experimental targeted therapies for 72 h. Scale bar is 50 μm. **(B,C)** Heatmap representation of the OMI index treatment effect size (Glass's Δ) for all patients at 72 h in organoids **(B)** and fibroblasts cultured with organoids. **(C)** “∧” indicates the patient lesion was diagnosed as PanIN. “~” indicates the patient lesion was diagnosed as ampullary cancer. *Glass's Δ ≥ 0.75. **(D)** Normalized density distributions of the OMI index of individual cells contain subpopulations with G+P treatment, but not ABT-263 + T treatment after 72 h in PC13 organoids. Bracketed number indicates number of subpopulations. **(E)** High-depth targeted cancer gene sequencing of PC13 organoids. Allele frequencies of ~50% for *KRAS* and 100% for *TP53* were found (black bars). Alterations with allele frequencies of 10–30% were detected (gray bars), but none of these alterations were pathogenic. **(F)** Cleaved caspase-3 staining of PC13 organoids shows differences in apoptosis between treatment conditions after 72 h of treatment. **(G)** Ki67 staining of PC13 organoids shows differences in proliferation between treatment conditions after 72 h. Each dot represents one organoid (mean ± SEM), and red indicates significant response to treatment. *p < 0.05 vs. control.

OMI was then used to track changes in metabolic heterogeneity in response to standard and experimental therapies in PC. After an establishment period, PC organoids were treated with a panel of standard and experimental therapies and imaged over a time course (Figure 4A). This drug panel included 5-FU and gemcitabine chemotherapy, along with an experimental combination of TAK-228 (mTORC1/2 inhibitor) and ABT-263 (Bcl-2 and Bcl-xL inhibitor). Additional standard drugs were tested on organoids from Patients PC6, PC13, and PC18 at later time points after patient treatment plans were obtained. A wide variety of OMI index responses were elicited across treatment conditions and patient samples. A heatmap of OMI index treatment effect size, calculated using Glass's Δ at 72 h post-treatment, shows significant inter-patient heterogeneity for drug response in organoids (Figure 4B; additional variables and time points in Supplementary Figure S4). In a subset of patient samples, a population of fibroblasts (co-cultured with organoids) migrated from the 3D matrix and adhered to the 2D glass coverslip. Heterogeneity in these fibroblasts has been shown in organoid models of murine PC (1, 66). A heatmap of OMI index treatment effect size, calculated using Glass's Δ at 72 h post-treatment, also shows inter-patient drug response heterogeneity in co-cultured fibroblasts (Figure 4C; Supplementary Figure S5). Repeated measurements of responses to drugs over 7 days demonstrate how OMI can track single-cell drug responses over time within organoids (Supplementary Figure S6) and co-cultured fibroblasts (Supplementary Figure S7). For example, response data show generally increased responses to TAK-228 and ABT-263 combination targeted therapy over time, while 5-FU remained ineffective for most patients. Viability and OMI were validated for human organoids by quantifying metabolic inhibition by cyanide, a known inhibitor of the electron transport chain (Supplementary Figure S8). The effects of cyanide on OMI endpoints agreed with previous reports (23).

### OMI Captures Non-Genetic Cellular Heterogeneity in Pancreatic Organoids

Additional analysis was performed on Patient PC13 organoids to evaluate the utility of OMI. The combination of gemcitabine and nab-paclitaxel was used in addition to the standard treatment panel on Patient PC13's organoids to mimic the treatment received prior to sample collection. Population density modeling was used to determine whether cellular subpopulations of metabolic response were present in organoids for each treatment condition at 72 h (Figure 4D; additional drugs in Supplementary Figure S9). Metabolic subpopulations were observed in controls and organoids treated with gemcitabine and nab-paclitaxel (G+P) combination. The patient responded poorly to this treatment prior to surgery and organoid generation.

High-depth targeted gene sequencing was performed on untreated Patient PC13 organoids to determine whether subclonal populations could be resolved to explain the metabolic heterogeneity (Figure 4E). A mutation in the *TP53* tumor-suppressor gene (stop-gain Gln165^*^) and a mutation in the *KRAS* oncogene (G12V) were found with allele frequencies of 100 and 52%, respectively, indicating a single population of cells with homogeneous driver mutations. Mutations with allele frequencies between 10 and 30% are indicative of potential subclonal populations (67). Only 4 alterations were found to occur within this range, with 3 of the 4 at frequencies just below the top of this range. None of the alterations identified are pathogenic or known to alter tumor biology, indicating that the sample was genetically homogenous with subpopulations likely related to metabolic changes and not differing mutation profiles. A subset of organoids was fixed 72 h post-treatment and stained using immunofluorescence for CC3 and Ki67 to quantify apoptosis and proliferation rates, respectively (Figures 4F,G, Supplementary Figure S10). Proliferation rates correlated with the changes in cell metabolism measured by OMI index (*p* < 0.001), but apoptosis rates did not (Supplementary Figure S11A). To determine if *in vivo* treatment response correlates with that seen in organoids, a patient-derived xenograft line was generated and athymic nude mice were treated with a combination therapy of gemcitabine and nab-paclitaxel (Supplementary Figure S12). Tumor growth was tracked by direct caliper measurement and an early reduction was observed over the first 7 days (*p* < 0.05); however, the effect was not sustained. This lack of response in the patient-derived xenograft is consistent with the heterogeneity in cell-level response observed with OMI for the same treatment of gemcitabine and nab-paclitaxel in the patient-derived organoid (Figure 4D).

### Heterogeneity of Drug Response in PC Organoids Agrees With Later Patient Recurrence During Adjuvant Therapy

**Figure 5:**
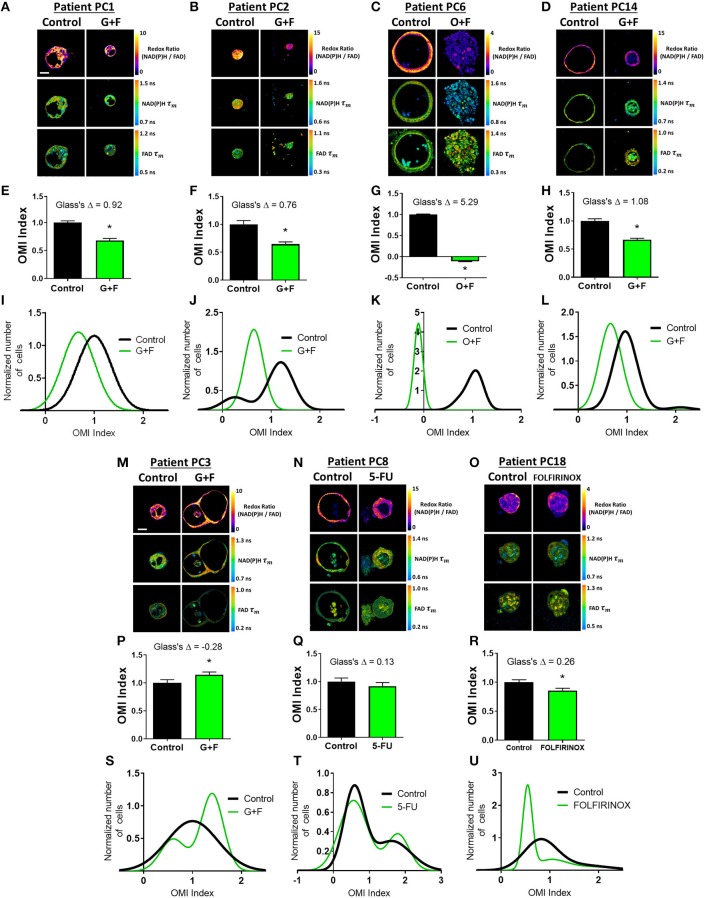
Organoid-based predictions of pancreatic cancer patient response to therapy. **(A–D)** Representative redox ratio, NAD(P)H τ_m_, and FAD τ_m_ images of pancreatic organoids from Patients PC1 **(A)**, PC2 **(B)**, PC6 **(C)**, and PC14 **(D)**, who are classified as predicted responders. Left columns indicate control organoids, and right columns indicate organoids treated with the same drugs as the patient adjuvant treatment. Scale bar is 50 μm. **(E–H)** The effect of the same drugs on the OMI index averaged across all cells in organoids derived from Patient PC1 **(E)**, PC2 **(F)**, PC6 **(G)**, and PC14 **(H)**. Error bars indicate mean ± SEM. *p < 0.0001. **(I–L)** Single-cell OMI index subpopulation analysis of treatment response in organoids from Patient PCI **(I)**, PC2 **(J)**, PC6 **(K)**, and PC14 **(L)**. **(M–O)** Representative redox ratio, NAD(P)H τ_m_, and FAD τ_m_ images of pancreatic organoids from Patients PC3 **(M)**, PC8 **(N)**, and PC18 **(O)**, who are classified as predicted non-responders. Left columns indicate control organoids, and right columns indicate organoids treated with the same drugs as the patient adjuvant treatment. **(P–R)** The effect of the same drugs on the OMI index averaged across all cells in organoids derived from Patient PC3 **(P)**, PC8 **(Q)**, and PC18 **(R)**. **(S–U)** Single-cell OMI index subpopulation analysis of treatment response in organoids from Patient PC3 **(S)**, PC8 **(T)**, and PC18 **(U)**.

Next, OMI of metabolic heterogeneity was assessed in PC patient-derived tumor organoids and compared to patient recurrence during adjuvant therapy. Clinical drug treatment efficacy was tracked and compared to the OMI prediction of drug response at 72 h post-treatment in patient-matched organoids (Figure 5). Four patients whose organoids exhibited a homogeneous response to the patient's prescribed therapy were classified as “predicted responders” (Figures 5A–L). Glass's Δ was calculated for each treatment's OMI index value in addition to statistical significance, and was found to be consistently >0.75. Cellular population density modeling of organoids from Patients PC1, PC2, PC6, and PC14 did not reveal distinct metabolic subpopulations of response (Figures 5I–L). In each case, only a single homogeneous population was observed. Fibroblasts co-cultured with organoids from Patients PC2 and PC14 also showed homogeneous response to the patient's prescribed therapy (Supplementary Figure S13).

Three patients (PC3, PC8, and PC18) whose organoids exhibited treatment response heterogeneity were classified as “predicted non-responders” (Figures 5M–U). On average, cells from Patient PC3 and PC8 organoids did not respond to the patient's prescribed treatment (*p* < 0.05 increase in OMI index and no significant change, respectively, Figures 5P,Q). Unlike PC3 and PC8, the OMI index of Patient PC18's organoid cells significantly decreased with treatment (*p* < 0.0001, Figure 5R); however, the response was heterogeneous (Figure 5U) which also predicts a poor outcome for the patient. The treatment effect size was small (Glass's Δ < 0.3) in organoids for all three patients, and all exhibited multiple subpopulations of tumor cells post-treatment, some of which overlapped completely with control distributions or contained OMI index values above control (Figures 5S–U). Fibroblasts co-cultured with organoids from Patient PC3 also showed a lack of response to gemcitabine and 5-FU, along with treatment-induced metabolic heterogeneity (Supplementary Figure S13).

**Figure 6:**
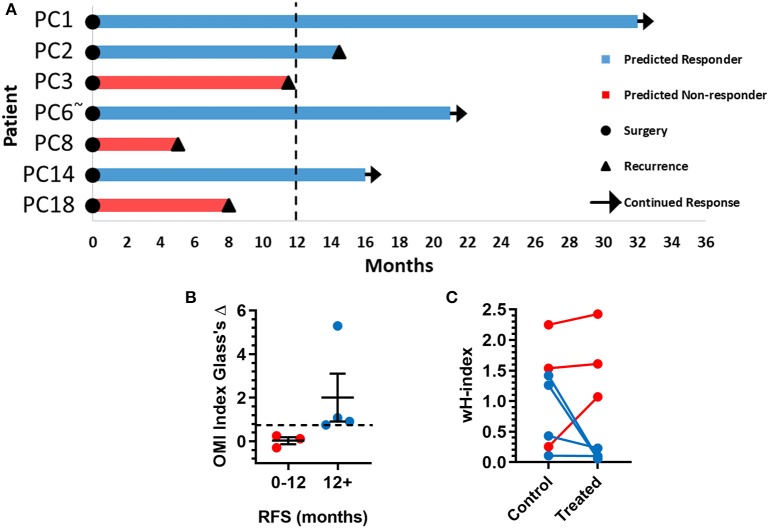
Pancreatic cancer patient clinical outcomes while on adjuvant therapy. **(A)** Swimmer plot indicating the number of months without recurrence following surgical resection of the tumor and adjuvant treatment. Patients are classified as predicted responders and non-responders based on organoid response profiles. Arrows indicate that the patient continues to survive without recurrence at the time of publication. “~” Indicates the patient's lesion was diagnosed as ampullary cancer. **(B)** Patients with RFS > 12 months had higher OMI index effect sizes at 72 h (Glass's Δ) than patients with RFS <12 months (mean± SEM). Dotted line represents proposed cutoff of Δ = 0.75. **(C)** Patients with RFS > 12 months show a decrease in wH-index with treatment compared to control organoids. Patients with RFS < 12 months show an increase in wH-index with treatment compared here to control organoids. Error bars not visible. *N* = 1,000 fits/group.

The time between surgical resection and the first evidence of recurrence, or recurrence-free survival (RFS) time, was plotted for these seven patients (Figure 6A). Patients PC3, PC8, and PC18, classified as predicted non-responders, experienced recurrences within 1 year. Patients PC1, PC2, PC6, and PC14, classified as predicted responders, each survived at least 1 year after surgery without recurrence. Patients with a RFS >12 months had a higher OMI index Glass's Δ in organoids at 72 h post-treatment than patients with a RFS <12 months (Figure 6B). The degree of heterogeneity, quantified by the wH-index, decreased in treated vs. control organoids in patients with a RFS >12 months, and increased in treated vs. control organoids in patients with a RFS <12 months (Figure 6C).

### OMI of BC Organoids Resolves Differential Sensitivities to Relevant Drug Treatments

**Figure 7:**
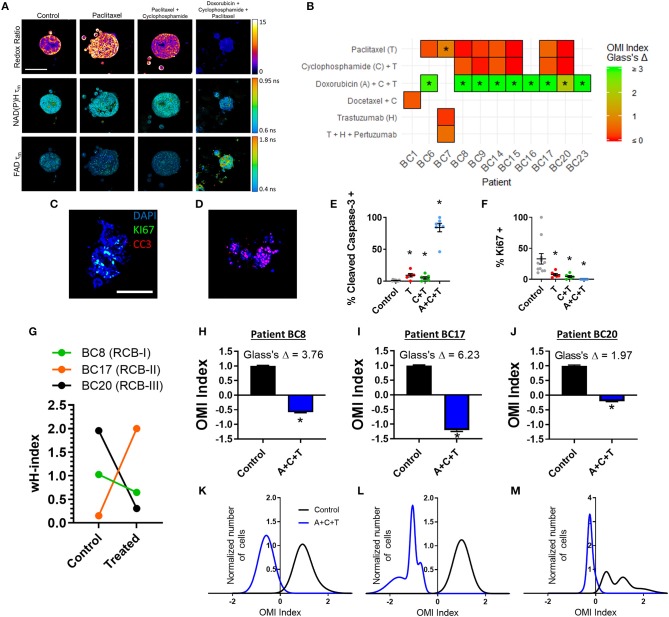
OMI of single-cell treatment response in patient-derived breast tumor organoids. **(A)** Representative images of the optical redox ratio, NAD(P)H τ_*m*_, and FAD τ_*m*_ organoids generated from Patient BC8 after 72 h of treatment. Scale bar = 100 μm. **(B)** Heatmap representation of the OMI index treatment effect size (Glass's Δ) at 72 h in organoids from each breast cancer patient. *Glass's Δ ≥ 0.75. 4-OOH cyclophosphamide (active metabolite) was used in place of cyclophosphamide. **(C,D)** Representative images of control **(C)** and A+C+T treated **(D)** BC8 organoids stained for Ki67 (green, proliferation), cleaved caspase-3 (red, apoptosis), and DAPI (blue, nuclei) after 72 h of treatment. Scale bar 100 μm. **(E)** Cleaved caspase-3 staining of organoids shows differences in apoptosis between treatment conditions after 72 h of treatment in BC8. **(F)** Ki67 staining of organoids shows differences in proliferation between treatment conditions after 72 h in BC8. Each dot represents one organoid (mean ± SEM). *p < 0.05 vs. control. **(G)** The effect of A+C+T treatment at 72 h on OMI index heterogeneity quantified by the wH-index in Patient BC8, BC17, and BC20 organoids. **(H–J)** The effect of 72 h A+C+T treatment on the OMI index averaged across all cells in organoids derived from Patient BC8 **(H)**, BC17 **(I)**, and BC20 **(J)**. Error bars indicate mean ± SEM. *p < 0.0001. Single cell OMI index subpopulation analysis of 72 h of A+C+T treatment response in organoids from Patient BC8 **(K)**, BC17 **(L)**, and BC20 **(M)**.

Next, treatment response in BC patient-derived organoids was assessed with OMI. A subset of 11 viable patient-derived BC organoid lines were treated with either the patient's prescribed neoadjuvant treatment, or a selection of standard chemotherapy drugs and imaged with OMI. For example, organoids derived from Patient BC8 were treated prior to OMI with the standard combination of paclitaxel, 4-OOH cyclophosphamide (the key active metabolite of cyclophosphamide) (51), and doxorubicin (A+C+T) to mimic the patient's prescribed neoadjuvant regimen (Figure 7A). As in pancreas organoids, treatment effect sizes were calculated using Glass's Δ on all OMI measurements in order to determine their magnitude (Figure 7B; additional variables and time points in Supplementary Figure S14). As in PC, OMI can also track single-cell drug response over time (Supplementary Figure S15). For example, Patient BC8 organoids exhibit an initial significant response to paclitaxel on days 1 and 2 (*p* < 0.05 vs. control), but the response is no longer present by day 3 (Supplementary Figure S15A). These organoids were also evaluated with traditional immunofluorescence cell markers (Figures 7C–F, Supplementary Figure S16). All three treatments caused both a significant increase in apoptosis (*p* < 0.05 vs. control, Figure 7E) and a significant decrease in proliferation (*p* < 0.05 vs. control, Figure 7F). Apoptosis correlated with changes in cellular metabolism measured by the OMI index (*p* < 0.05), but proliferation rates did not (Supplementary Figure S11B).

For three BC patients (BC8, BC17, BC20), viable organoids were grown and treated with A+C+T for 72 h to mirror the patient's neoadjuvant treatment regimen. The change in the degree of heterogeneity in the patient organoids after 72 h of A+C+T was quantified using the wH-index (Figure 7G). A breast pathologist (SMM) assigned Patient BC8 into residual cancer burden category I (RCB-I) following neoadjuvant treatment with A+C+T, indicating minimal residual disease (68). Patient BC17 was assigned to RCB-II, indicating moderate residual disease, while Patient BC20 was assigned to RCB-III, indicating extensive residual disease (68). On average, the OMI index of cells from all three patients decreased with A+C+T treatment with large effect sizes (*p* < 0.0001, Glass's Δ > 1.9, Figures 7H–J), but OMI index heterogeneity was altered to varying degrees (Figures 7K–M).

### Treatment Response Heterogeneity Complements Glass's Δ

Finally, changes in heterogeneity measurements and mean FLIM measurements with treatment in both cancer types were analyzed to determine the relationship between these variables and a given treatment's Glass's Δ. A map of bivariate correlations indicates that a vast majority of treatment-response measurements correlate (*R*^2^ > 0.5) with, at most, one other measurement (Supplementary Figure S17). This suggests that the measurements in this set could each capture unique information about the effect of a particular drug treatment. Multivariate regression was performed with OMI index Glass's Δ or change in wH-index as the dependent variable, and heterogeneity variables or mean FLIM variables as the set of independent variables (Supplementary Table S3, heterogeneity and mean FLIM variables defined in Supplementary Figure S17). OMI index Glass's Δ is better captured by the mean FLIM treatment response variables, while the change in wH-index with treatment better captured the heterogeneity treatment response variables. Of note, the mean FLIM variables alone only explain 4% of the variance in the change in wH-index across all treatments and both cancer types (adjusted *R*^2^ = 0.04). Additionally, changes in heterogeneity variables with treatment explain more variance in OMI index Glass's Δ in BC (adjusted *R*^2^ = 0.76) than in PC (adjusted *R*^2^ = 0.34).

## Discussion

Organoids can be used for drug screens directly on patient cells, which could enable rational treatment planning for individual patients (1–3). Organoids also provide a platform to discover new drugs and drug combinations to treat PC patients, who currently suffer from a severe lack of effective treatment options. Existing methods to evaluate drug response in organoids ignore cellular heterogeneity, which can lead to patient relapse. Thus, our group developed OMI as a single-cell analysis tool to detect minority subpopulations of drug-resistant cells existing within living organoids that would otherwise appear responsive. We have previously shown that OMI detects subpopulations of drug response in murine PC organoids, patient-derived colorectal cancer organoids, and patient-derived BC organoids (1, 35, 38). Here, we analyze and characterize baseline heterogeneity in organoids derived from individual PC and BC patients, and investigate for the first time whether OMI measurements of early drug response heterogeneity in organoids can capture meaningful treatment responses.

Three independent OMI endpoints [redox ratio, NAD(P)H τ_*m*_, and FAD τ_*m*_] were quantified at the single-cell level to assess the metabolic heterogeneity present in each patient's organoids at baseline. Each OMI endpoint captures unique metabolic information (23), and quantitatively combining these independent measurements into one OMI index provides a technique to evaluate the overall metabolic state of each cell. We first analyzed the sources of cellular metabolic variability across all samples to determine whether single-cell approaches that look within organoids are necessary to capture intra-tumor metabolic heterogeneity. It was first determined using linear regression that only a fraction of cellular heterogeneity could be explained by differences between organoids within a patient-derived line (16–36%, Figure 2). This suggests that techniques measuring intra-patient heterogeneity at the organoid level fail to capture the full extent of heterogeneity present in a sample, and that single-cell techniques such as OMI are required. Our results also indicate that single-cell techniques may be especially important in BC. A large portion of the overall cellular variation between all cells was explained by variation between patients (39–78%, Figure 2), highlighting the need for personalized medicine tools such as OMI that account for the unique metabolic profiles of individual patients. Finally, comparison with cytologic variability in BC tissue sample histology provided evidence that OMI of patient-derived organoids can accurately capture the heterogeneity present in the original tumor *in vivo* (Supplementary Figure S2).

The relationship between baseline intra-organoid heterogeneity, metabolism, and organoid morphology was also investigated (Figure 3). Organoids from murine PC models have been shown to deviate into two morphological types with unique metabolic characteristics measured by OMI (1), but this has not been studied in patient-derived organoids or in terms of intra-organoid heterogeneity measurements. In this study, patient-derived organoids were observed to either form a thin spherical layer of cells surrounding a hollow lumen, or remain in a solid morphology of cells throughout and no lumen. The former was expected, as epithelial structures such as mammary glands and pancreatic ducts are made up of monolayers of cells enclosing a central lumen (69). Differences in metabolism and heterogeneity between hollow and solid organoids were also expected, as heterogeneity-driving gradients of oxygen and nutrients are known to form within solid tumor spheroids (70). While solid PC organoids were more heterogeneous than hollow PC organoids in terms of OMI index standard deviation and redox ratio CV, other variables did not show this trend, suggesting that factors beyond diffusion gradients may influence intra-organoid heterogeneity. PC organoids also formed hollow lumens more often than BC organoids. Relationships between organoid morphology and metabolism may be specific to the microenvironment, 3D architecture, and signaling properties of the epithelial organ of origin. Future studies to track the development of both heterogeneity and morphology within individual organoids could further elucidate these relationships. Resistance to apoptotic drugs correlates with 3D tissue organization and lumen formation (71), suggesting that a greater understanding of the structural forces influencing tumor heterogeneity could lead to improved treatment planning and new treatment strategies.

Early metabolic changes were quantified in response to panels of standard drugs and experimental targeted therapies in PC organoids (Figure 4). There is an unmet need for this technology, which would allow oncologists to quickly determine if a patient would benefit from experimental targeted therapies over standard chemotherapies, rather than waiting for standard chemotherapy to fail while exposing the patient to unnecessary toxicities. Drug response was also evaluated using OMI in patient-derived fibroblasts, which grew along with organoids for most PC patients. The dense fibrotic extracellular matrix surrounding pancreatic tumors can hinder drug delivery by reducing blood flow and raising interstitial fluid pressure (72, 73). Thus, it may be vital to evaluate whether drugs can target both the tumor and its stromal microenvironment to enhance delivery (11, 12). For example, Patient PC18's organoids showed response to the combination of TAK-228 and ABT-263 (Glass's Δ ≥ 0.75) at 72 h while co-cultured Patient 18 fibroblasts did not, suggesting that this drug regimen could successfully kill tumor cells but the drugs may not be able to penetrate the fibroblast-rich tumor microenvironment. This highlights a need for technologies such as OMI that can assess multiple cell types in 3D organoids to discover new treatment strategies that target both a tumor and its stroma.

Our group has shown that OMI non-invasively distinguishes unique groups of cells by their metabolic properties in human BC (35, 37), human head and neck cancer (36), human colorectal cancer (38), and murine PC (1). Here, we examined Patient PC13 organoid cells using OMI to evaluate whether OMI could distinguish cells with distinct drug responses in human PC (Figure 4, Supplementary Figure S9). Our results suggest that a drug-resistant cell subpopulation that persisted throughout the patient's neoadjuvant treatment was captured in the organoids. Accordingly, pathology of the patient's resected tumor indicated a poor response to gemcitabine with nab-paclitaxel. Conversely, the experimental combination of TAK-228 and ABT-263 induced a homogenous response in Patient PC13 organoids. This suggests that this therapy may have been a beneficial alternative for Patient PC13. One month after surgery, metastasis in the liver was detected by ultrasound, and the patient died <2 weeks later, emphasizing the need for technology that can rapidly evaluate drug sensitivity in patient cells.

The combination of gemcitabine and nab-paclitaxel was evaluated *in vivo* in a PDX line established from Patient PC13 organoids. A small but transient response in average tumor growth was detected (Supplementary Figure S12). This poor response is in agreement with the heterogeneous effect found in organoids using OMI. While the PDX model accurately captured the presence of drug resistance in Patient PC13's cells, it required months to first establish the PDX line, expand it, and then assess a time course of treatment. While PDX models are an important tool, our studies show that drug screens on organoids can provide more detailed response information with increased cost effectiveness in a clinically meaningful time frame.

OMI of organoids generated from tissues collected at surgery agreed with treatment outcomes for PC patients on adjuvant therapy (Figures 5, 6). Treatment response in organoids was evaluated with metabolic heterogeneity and the Glass's Δ of the OMI index. For Patients PC1, PC2, PC3, PC6, PC8, PC14, and PC18, OMI of organoid heterogeneity predicted whether the patient survived 1 year post-surgery without recurrence. Based on data from this initial patient cohort, a proposed decrease in OMI index (effect size cutoff of 0.75) along with a treatment-induced decrease in wH-index could classify patients as predicted responders vs. non-responders. Overall, our results suggest that early metabolic responses in pancreatic organoids can successfully capture the response of tumor cells *in vivo* that are not removed during surgery. In all cases, sufficient organoids were generated to assess the patient's prescribed drug treatment in addition to multiple alternative drug options. This indicates that OMI of organoids could support drug discovery and development within diverse patient populations. While many other factors beyond tumor cell treatment response affect PC survival in the adjuvant setting (i.e., surgical margins, stage, grade, etc.), OMI of organoids could identify drug resistance and eliminate objectively poor drug options.

OMI successfully tracked single-cell drug response in a subset of 11 BC patient-derived organoids (Figure 7). A+C+T treatment resulted in a large Glass's Δ on the average OMI index across organoid cells in all patients tested, supporting the presently widespread use of this treatment in BC patients. For a subset of three patients that were given neoadjuvant treatment (BC8, BC17, BC20), we evaluated the potential of OMI to measure cellular heterogeneity in organoids derived from diagnostic core needle biopsies in response to the patient's prescribed treatment. BC20 had the smallest OMI index effect size and greatest heterogeneity pre-treatment, resulting in the worst response to neoadjuvant treatment (RBC-III). Although the sample size for these BC response studies is small, this indicates BC patient response to neoadjuvant treatment is related to both treatment effect and heterogeneity in organoids.

We next used our data set of organoid treatment groups from all patients and time points to determine whether treatment-induced metabolic heterogeneity adds complimentary information to the average metabolic change with treatment (*N* = 401, Supplementary Table S3, Supplementary Figure S17). A variety of methods for quantifying cellular heterogeneity were incorporated in this analysis, including the effects of treatment on wH-index, QE, KS, OL, and the percentage of cellular variation explained by organoid variation. Results indicate that OMI index Glass's Δ and the wH-index capture independent dynamics of response and provide complementary information. Therefore, single-cell drug response measurement techniques such as OMI could improve cancer treatment planning and drug development compared to drug response averaged across all cells.

Organoids offer a compelling platform for the interrogation of a variety of drugs *ex vivo*. OMI has many benefits over existing methods to assess drug response in organoids because it is non-destructive, label-free, and it measures unique features of cell metabolism. NAD(P)H and FAD are involved in hundreds of metabolic reactions, so OMI provides a holistic picture of cell metabolism by quantifying the redox state and enzyme binding activity of these ubiquitous co-enzymes. Current methods require exogenous labels, involve fixation of cells, and/or require sample dissociation. Additionally, OMI can measure response on the single-cell level to assess heterogeneity as quickly as 24 h post-treatment and can track dynamic responses over time. Existing methods for screening drugs and measuring their efficacy in organoids generally report the amalgamated effects of individual cells, ignoring heterogeneity. This can lead to the inadvisable selection of drugs for which a subpopulation of resistant cells may continue to thrive in the tumor. In this study, we used these optical imaging tools to show that organoid drug screens can assess heterogeneous drug responses in multiple cancer types and subtypes within a clinically meaningful time frame. Taken together, OMI of organoids is a sensitive, high-throughput tool to assess single-cell metabolic response, which could improve patient outcomes and enable new drug discovery.

## Data Availability Statement

The original contributions presented in the study are publicly available. This data can be found here: NCBI SRA (https://www.ncbi.nlm.nih.gov/bioproject/PRJNA612905). All other experimental data required to reproduce the findings from this study will be made available upon request.

## Ethics Statement

The studies involving human participants were reviewed and approved by Institutional Review Boards at the University of Wisconsin-Madison and the Medical College of Wisconsin (IRB# 2018-1104, IRB# UW14035). The patients/participants provided their written informed consent to participate in this study. This animal study was reviewed and approved by UW-Madison Institutional Animal Care and Use Committee.

## Author Contributions

JS, DD, and MS designed the study. CS, CP, DP, KE, AC, and RG-V contributed to the preparation of patient organoids and xenografts. JS performed experiments and wrote the manuscript with input from all authors. JS and CW analyzed the data. DD, CS, MB, AP, IM, MK, and ST helped identify potential donors and procure tissue samples. SM and KM analyzed patient pathology slides. CW designed the statistical analyses. DD analyzed genetic sequencing data. All authors contributed to manuscript revision.

## Conflict of Interest

The authors declare that the research was conducted in the absence of any commercial or financial relationships that could be construed as a potential conflict of interest.
