# Supplementary material for: Metabolic Heterogeneity in Patient Tumor-Derived Organoids by Primary Site and Drug Treatment
Source: Front Oncol. 2020 May 15;10:553. doi: 10.3389/fonc.2020.00553 (PMC7242740; doi:10.3389/fonc.2020.00553)
Supplement: Supplementary file 1 [file Image_1.PDF]

Supplemental figures

Supplementary Table S1
Characteristics of patient tissue samples acquired from surgical resection of pancreatic lesions. RFS, recurrence-free survival. * = recurrence has occurred, ^ = thawed from frozen stocks, # = organoids grew but were not passaged due to bacterial contamination

Patient #	Drug treatment before surgery	Neoadjuvant Treatment Response	Post-treatment Diagnosis	Cytologic Variability	Residual Tumor Size	Stage	Viable Organ-oids	Organoid Establishment Time (days)	Drug treatment after surgery	Known RFS (months)
PC1	5-FU	Partial response, score 2	PDAC, well-differentiated	Moderate	3.5x1.8x1.7 cm	ypT3 ypN0	Yes	10	Gemcitabine + 5-FU	>32
PC2	None	N/A	PDAC, poorly-differentiated	High	4.8x3.5x2 cm	pT3 pN1	Yes	4	Gemcitabine + 5-FU	14.5*
PC3	5-FU	Poor or no response, score 3	PDAC, poorly-differentiated	High	2x2x1.5 cm	ypT3 ypN1	Yes	6	Gemcitabine + 5-FU	11.5*
PC4	None	N/A	Mucinous adenocarcinoma	N/A	4x3x2.9 cm	pT3 pN1	No	N/A	Gemcitabine + 5-FU	>26
PC5	FOLFIRINOX	Complete response, score 0	No residual cancer	N/A	N/A	ypT0 ypN0	Yes	5	None	N/A
PC6	None	N/A	Ampullary adenocarcinoma, moderately-differentiated	High	2x2x1.2 cm	pT2 pN1	Yes	25^	Oxaliplatin + 5-FU	>21
PC7	None	N/A	PDAC, moderately-differentiated (arising in IPMN)	N/A	0.5 cm	pT1 pN0	No	N/A	None	>14
PC8	Gemcitabine + nab-paclitaxel	Partial response, score 2	PDAC, moderately-differentiated	High	3.0 cm	ypT3 ypN1	Yes	9	5-FU	5*
PC9	Gemcitabine + nab-paclitaxel + FOLFIRINOX	Poor or no response, score 3	PDAC, moderately-differentiated	N/A	3.1x2.5x2.3 cm	ypT4 ypN1	No	N/A	None	4*
PC10	None	N/A	PDAC, moderately-differentiated	N/A	3.5x2x2 cm	pT3 pN1	No	N/A	Unknown	2*
PC11	None	N/A	Chronic pancreatitis and PanIN-1	N/A	N/A	N/A	Yes	9	None	N/A
PC12	None	N/A	PDAC, poorly-differentiated	N/A	1.2x1.1x1.1 cm	pT1 pN1	Yes	N/A#	FOLFIRINOX	2*
PC13	Gemcitabine + nab-paclitaxel	Poor or no response, score 3	Undifferentiated (anaplastic) carcinoma of pancreas	Low	4.9x4.3x4.1 cm	ypT3 ypN1	Yes	11	None	0.5*
PC14	None	N/A	PDAC, moderately-differentiated	Low	3.5 cm	pT3 pN1	Yes	5	Gemcitabine + 5-FU	>16
PC15	None	N/A	PDAC, moderately-differentiated	N/A	3x2.5x2.5 cm	pT2 pTN1	No	N/A	Gemcitabine + 5-FU	>15
PC16	Gemcitabine + nab-paclitaxel	Partial response, score 2	Ampullary adenocarcinoma, well-differentiated	N/A	2.5x2.5x1.4 cm	ypT3b ypN1	No	N/A	None	>14
PC17	FOLFIRINOX	Partial response, score 2	PDAC, well-differentiated	Moderate	2.0x2.0x1.5 cm	ypT2 ypN0	Yes	12	None	4*
PC18	None	N/A	PDAC, poorly-differentiated	High	4.7x4.3x3.5 cm	pT3 pN2	Yes	11	FOLFIRINOX	8*
PC19	Gemcitabine + nab-paclitaxel	Partial response, score 2	PDAC, moderately-differentiated	Low	2.0x1.6x1.2 cm	ypT1C ypN0	Yes	33	None	>4
PC20	FOLFIRINOX + Gemcitabine	Partial response, score 2	PDAC, moderately-differentiated	N/A	1.5 cm	ypT1 ypN0	No	N/A	None	>2
PC21	FOLFIRINOX	Poor or no response, score 3	PDAC, moderately to poorly-differentiated	High	3.0x2.2x2.0 cm	ypT2 ypN0	Yes	12	None	3*
PC22	FOLFIRINOX + Gemcitabine	Partial response, score 2	PDAC, moderately-differentiated	N/A	2.5 cm	ypT2 ypN0	No	N/A	None	>1

Supplementary Table S2
Characteristics of patient tissue samples acquired from core needle biopsy of breast lesions. RCB, residual cancer burden

Patient #	Receptor Status	Patient Ki67%	Patient ER+ %	Grade	Cytologic Variability	Viable Organoids?	Establishment time (days)	Neoadjuvant Treatment Regimen	RCB score	RCB class
BC1	ER+/PR+/HER2-	15	95	2	Moderate	Y	56	None	-	-
BC2	ER+/PR-/HER2-	95	30	3	N/A	N	N/A	None	-	-
BC3	ER+/PR+/HER2+	90	70	3	N/A	N	N/A	None	-	-
BC4	ER+/PR+/HER2-	10	90	2	N/A	N	N/A	None	-	-
BC5	ER+/PR+/HER2-	10	90	2	N/A	N	N/A	None	-	-
BC6	ER+/PR+/HER2-	15	80	2	N/A	Y	26	None	-	-
BC7	ER-/PR-/HER2+	15	-	3	Moderate	Y	51	Paclitaxel + Trastuzumab + Pertuzumab	N/A, treatment ended early	
BC8	TN	80	-	3	High	Y	17	Doxorubicin + Cyclophosphamide + Paclitaxel	0.883	I
BC9	ER+/PR+/HER2-	20	95	2	Low	Y	27	None	-	-
BC10	ER+/PR+/HER2-	15	95	2	N/A	N	N/A	None	-	-
BC11	ER+/PR+/HER2-	75	95	3	N/A	N	N/A	None	-	-
BC12	ER+/PR-/HER2+	72	95	2	N/A	N	N/A	None	-	-
BC13	ER+/PR+/HER2-	70	95	2	N/A	N	N/A	Anastrozole	4.141	III
BC14	ER+/PR+/HER2-	10	95	2	Low	Y	68	None	-	-
BC15	ER+/PR-/HER2-	80	90	3	Low	Y	34	None	-	-
BC16	ER+/PR+/HER2-	10	100	2	Low	Y	46	None	-	-
BC17	TN	70	-	2	High	Y	24	Doxorubicin + Cyclophosphamide + Paclitaxel	1.965	II
BC18	TN	70	-	3	N/A	N	N/A	None	-	-
BC19	ER+/PR+/HER2-	80	80	2	N/A	N	N/A	None	-	-
BC20	TN	60	-	3	Low	Y	28	Doxorubicin + Cyclophosphamide + Paclitaxel	3.39	III
BC21	ER+/PR+/HER2-	80	95	2	N/A	Y	58	None	-	-
BC22	ER+/PR+/HER2-	10	95	2	Low	Y	49	None	-	-
BC23	ER+/PR+/HER2-	12	95	2	N/A	Y	27	None	-	-
BC24	ER+/PR+/HER2+	50	95	3	N/A	N	N/A	Doxorubicin + Cyclophosphamide + Paclitaxel + Trastuzumab + Pertuzumab	*pending*	

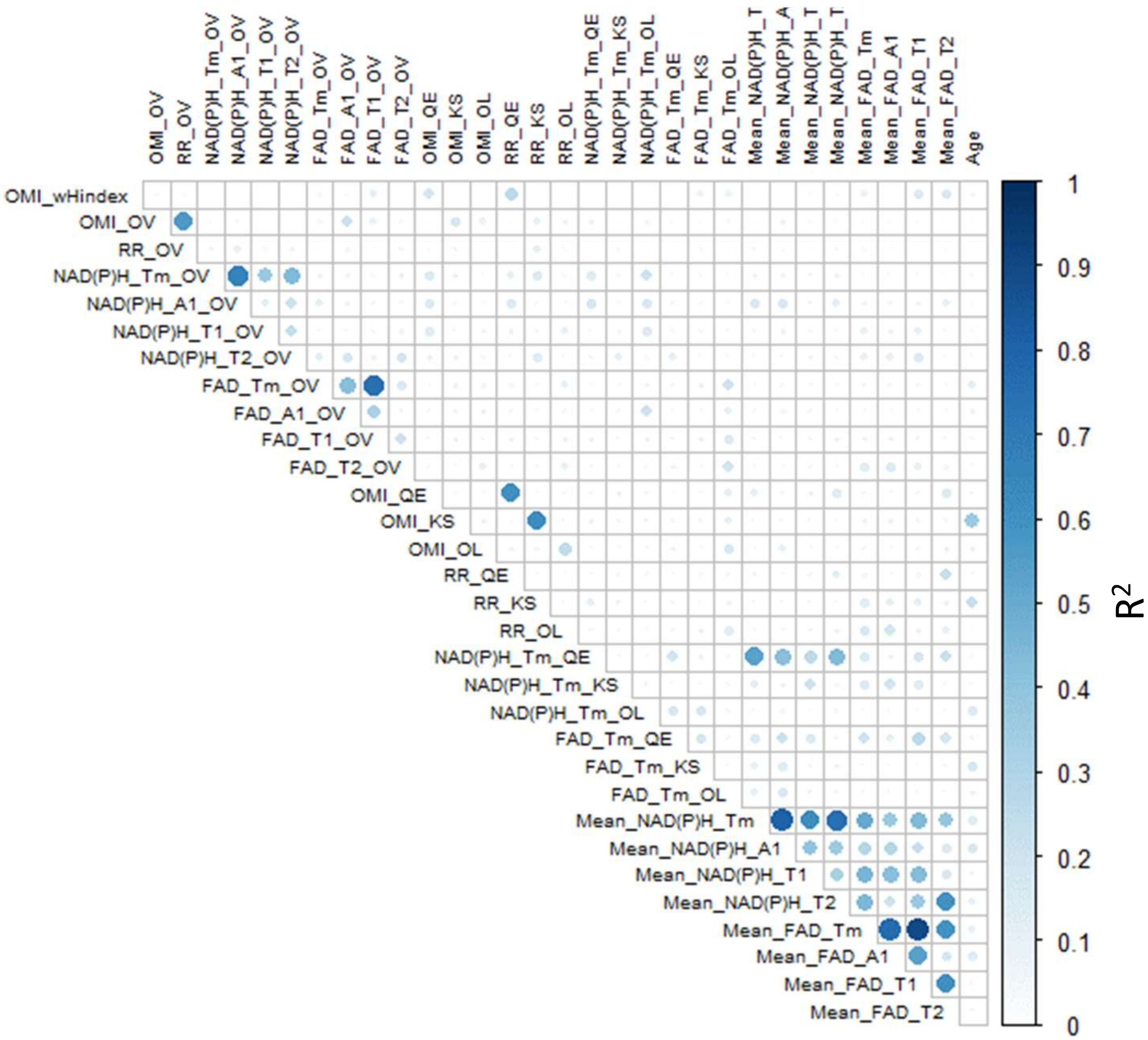

Supplementary Figure S1. Bivariate correlations between patient baseline heterogeneity and mean metabolism variables. R^2 strengths of correlations between all heterogeneity measurements and mean metabolic measurements, across all BC and PC patients combined (N=26). Untreated organoids at first imaging time point (baseline) only. OV = percentage of cellular variation explained by organoid variation, KS = Kolmogorov-Smirnov distance, QE = quadratic entropy, OL = outlier percentage, OMI = OMI index, RR = redox ratio.

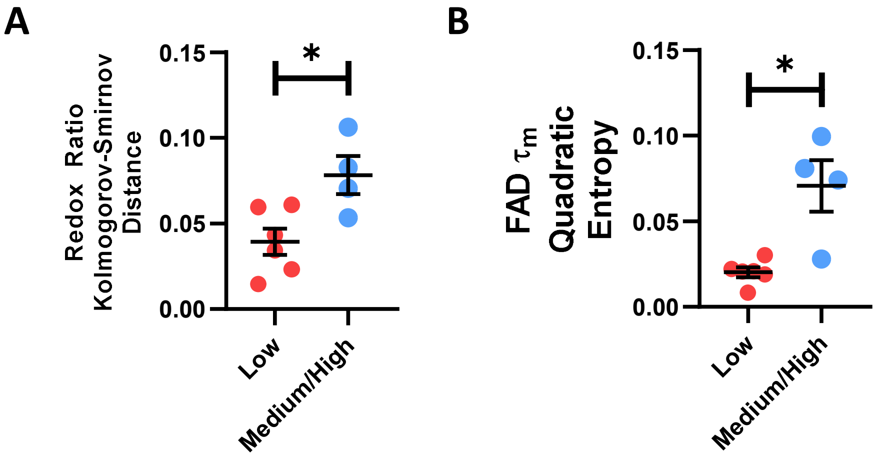

Supplementary Figure S2. Organoid OMI baseline heterogeneity variables agree with cytologic variability in the original breast biopsy tissue. An independent breast pathologist used H+E stained slides of breast biopsy from each patient to categorize cytologic variability as either low, medium, or high. **A,** The KS of the distribution of single-cell redox ratios was significantly higher in patients with medium or high cytologic variability vs. low. * $p<0.05$. Each dot corresponds to one patient. **B,** The QE of the distribution of single-cell FAD mean lifetimes was significantly higher in patients with medium or high cytologic variability vs. low.

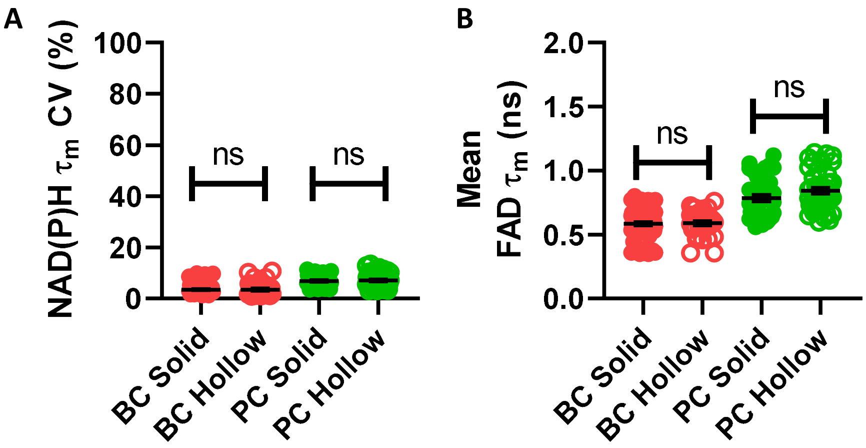

Supplementary Figure S3. Metabolic consistencies between hollow and solid organoids. A, Intra-organoid cellular metabolic heterogeneity was consistent between cancer types and organoid morphologies in terms of NAD(P)H τ_m CV. ns = p>0.05. **B,** Intra-organoid mean FAD τ_m CV was consistent between cancer types and organoid morphologies.

A

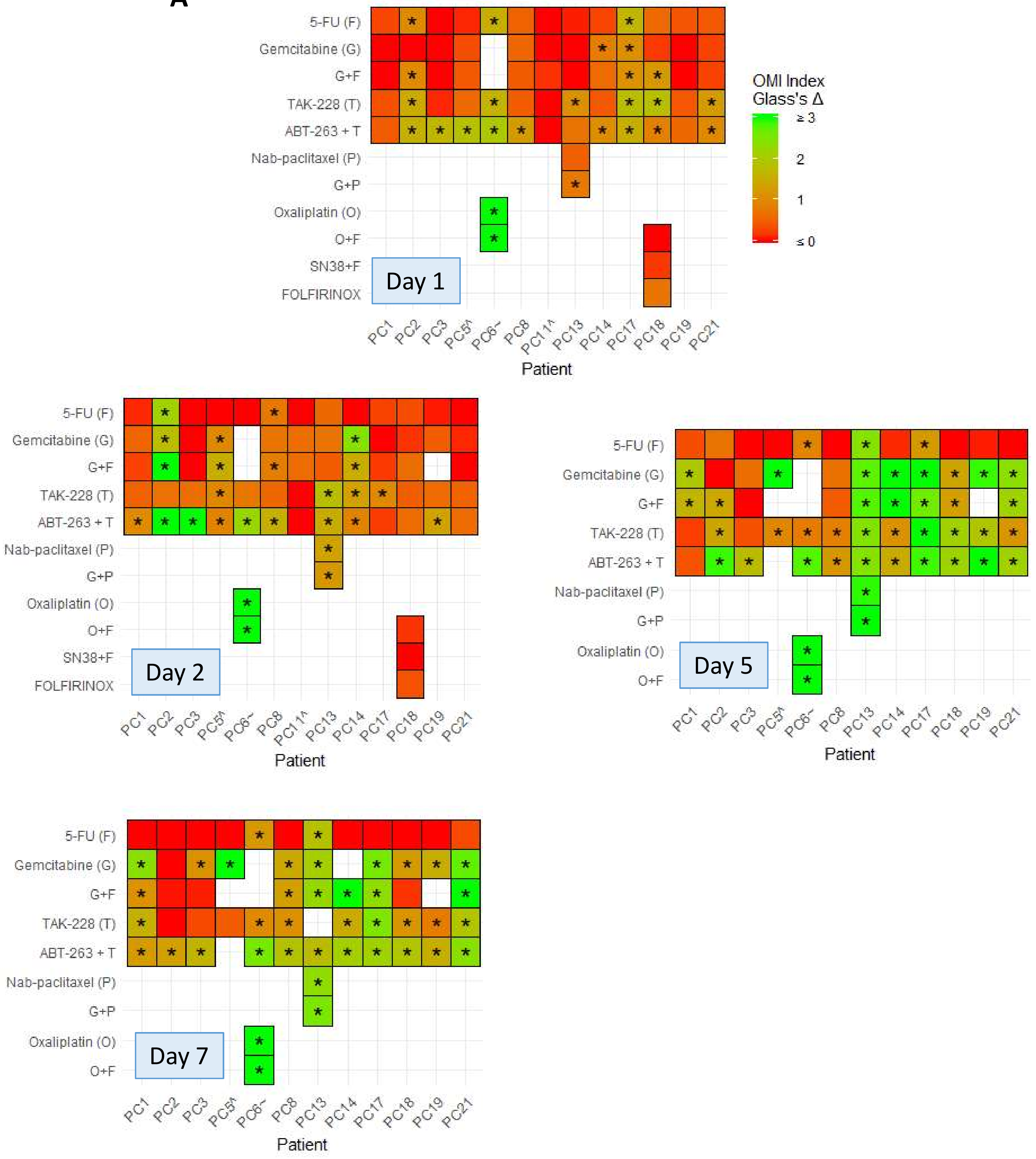

5-FU (F)
Gemcitabine (G)
G+F
TAK-228 (T)
ABT-263 + T
Nab-paclitaxel (P)
G+P
Oxaliplatin (O)
O+F
SN38+F
FOLFIRINOX
Day 1
PC1 PC2 PC3 PC5^ PC6~ PC8 PC11^ PC13 PC14 PC17 PC18 PC19 PC21
Patient
OMI Index
Glass's Δ
≥ 3
2
1
≤ 0
Day 2
Day 5
PC1 PC2 PC3 PC5^ PC6~ PC8 PC13 PC14 PC17 PC18 PC19 PC21
Day 7

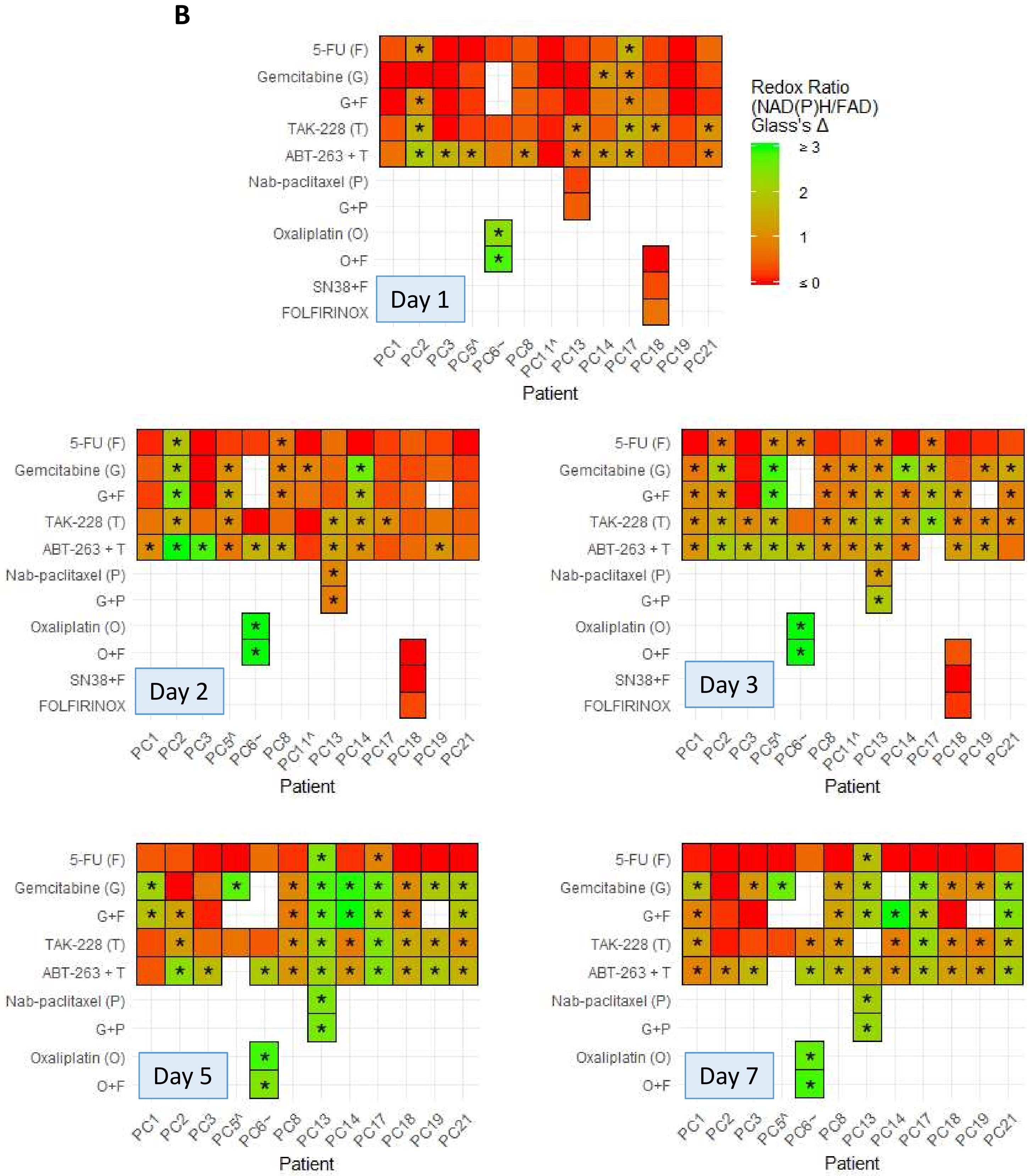
B
Day 1
Day 2
Day 3
Day 5
Day 7
5-FU (F)
Gemcitabine (G)
G+F
TAK-228 (T)
ABT-263 + T
Nab-paclitaxel (P)
G+P
Oxaliplatin (O)
O+F
SN38+F
FOLFIRINOX
PC1
PC2
PC3
PC5^
PC6~
PC8
PC11^
PC13
PC14
PC17
PC18
PC19
PC21
Patient
Redox Ratio (NAD(P)H/FAD) Glass's Δ
≥ 3
2
1
≤ 0

C

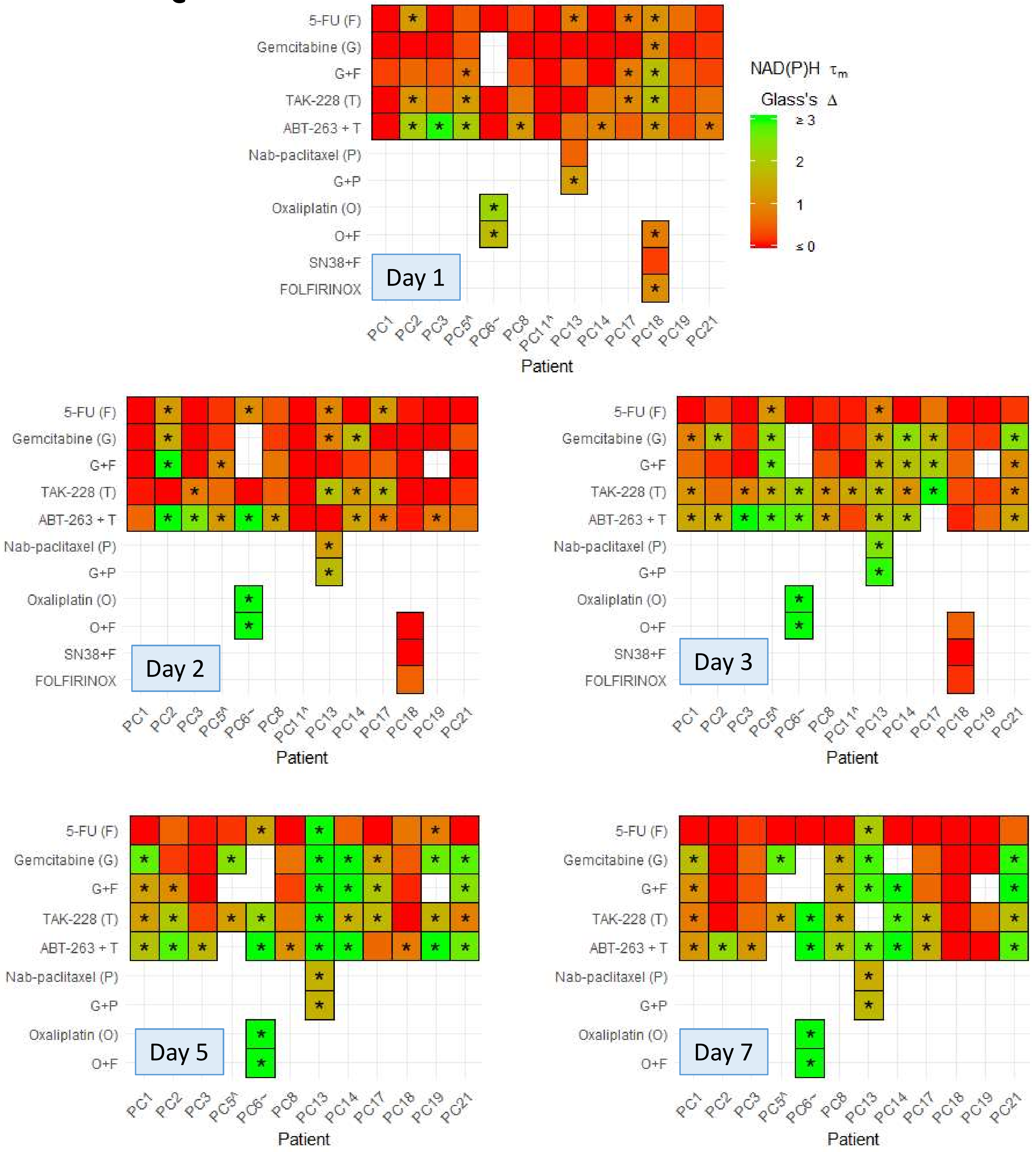

5-FU (F)
Gemcitabine (G)
G+F
TAK-228 (T)
ABT-263 + T
Nab-paclitaxel (P)
G+P
Oxaliplatin (O)
O+F
SN38+F
FOLFIRINOX
Day 1
PC1 PC2 PC3 PC5^ PC6~ PC8 PC11^ PC13 PC14 PC17 PC18 PC19 PC21
Patient
NAD(P)H τ_m
Glass's Δ
≥ 3
2
1
≤ 0
Day 2
Day 3
Day 5
PC1 PC2 PC3 PC5^ PC6~ PC8 PC13 PC14 PC17 PC18 PC19 PC21
Day 7

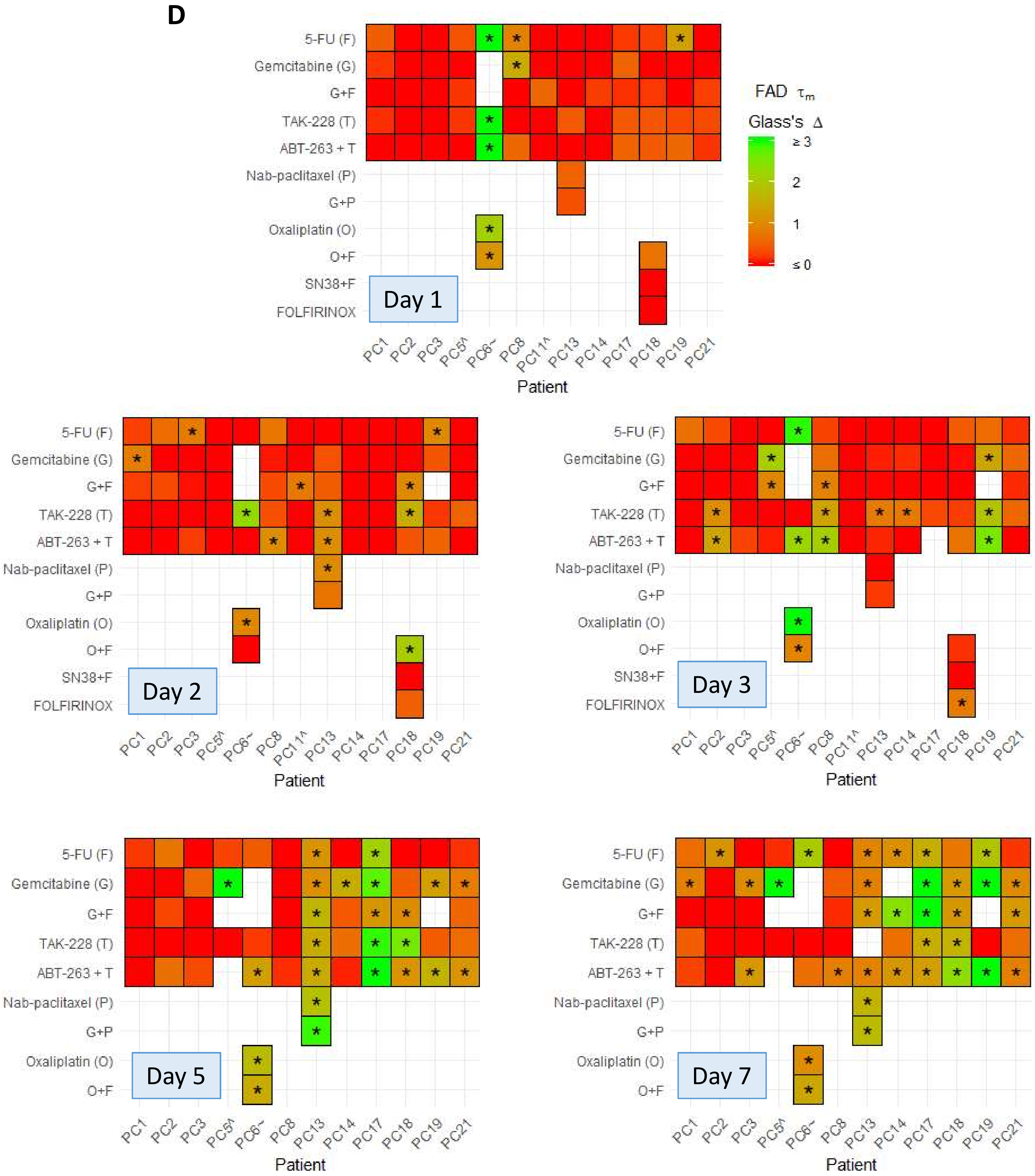
D
FAD τm
Glass's Δ
≥ 3
2
1
≤ 0
5-FU (F)
Gemcitabine (G)
G+F
TAK-228 (T)
ABT-263 + T
Nab-paclitaxel (P)
G+P
Oxaliplatin (O)
O+F
SN38+F
FOLFIRINOX
Day 1
Day 2
Day 3
Day 5
Day 7
PC1
PC2
PC3
PC5^
PC6~
PC8
PC11^
PC13
PC14
PC17
PC18
PC19
PC21
Patient

Supplementary Figure S4. Effect sizes of drug treatment on individual OMI endpoints in patient-derived pancreas organoids. A-D, Heatmap representation of the treatment effect size (Glass's Δ) at each time point for the OMI index (**A**), redox ratio (**B**), NAD(P)H τ_m (**C**), and FAD τ_m (**D**). '^' indicates the patient lesion was diagnosed as PanIN. '~' indicates the patient lesion was diagnosed as ampullary cancer. * Glass's $\Delta \geq 0.75$ vs. control.

A

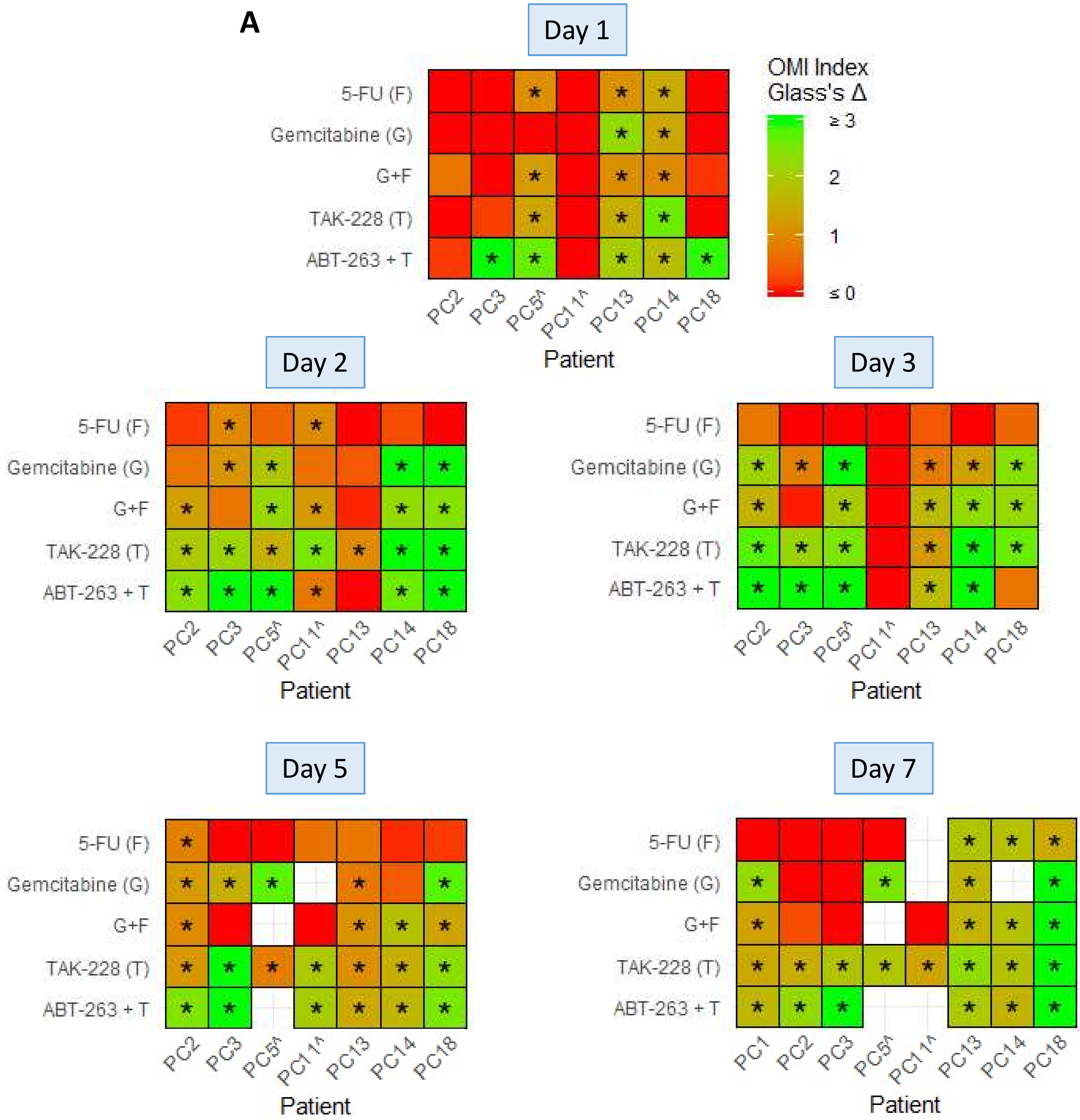

Day 1
Day 2
Day 3
Day 5
Day 7
5-FU (F)
Gemcitabine (G)
G+F
TAK-228 (T)
ABT-263 + T
PC1
PC2
PC3
PC5^
PC11^
PC13
PC14
PC18
Patient
OMI Index
Glass's Δ
≥ 3
2
1
≤ 0

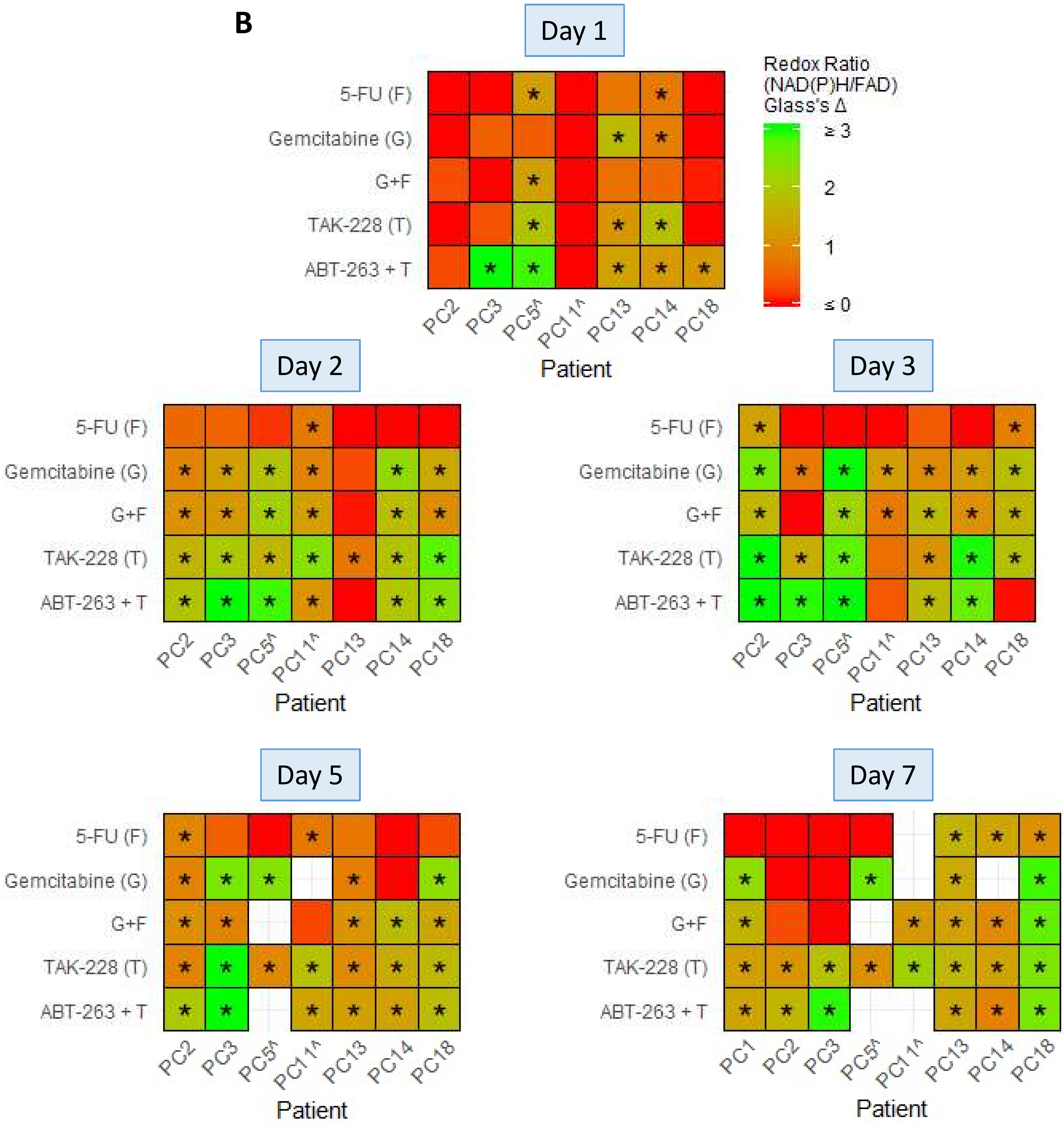
B
Day 1
Redox Ratio
(NAD(P)H/FAD)
Glass's Δ
≥ 3
2
1
≤ 0
5-FU (F)
Gemcitabine (G)
G+F
TAK-228 (T)
ABT-263 + T
PC2
PC3
PC5^
PC11^
PC13
PC14
PC18
Patient
Day 2
Day 3
Day 5
Day 7
PC1

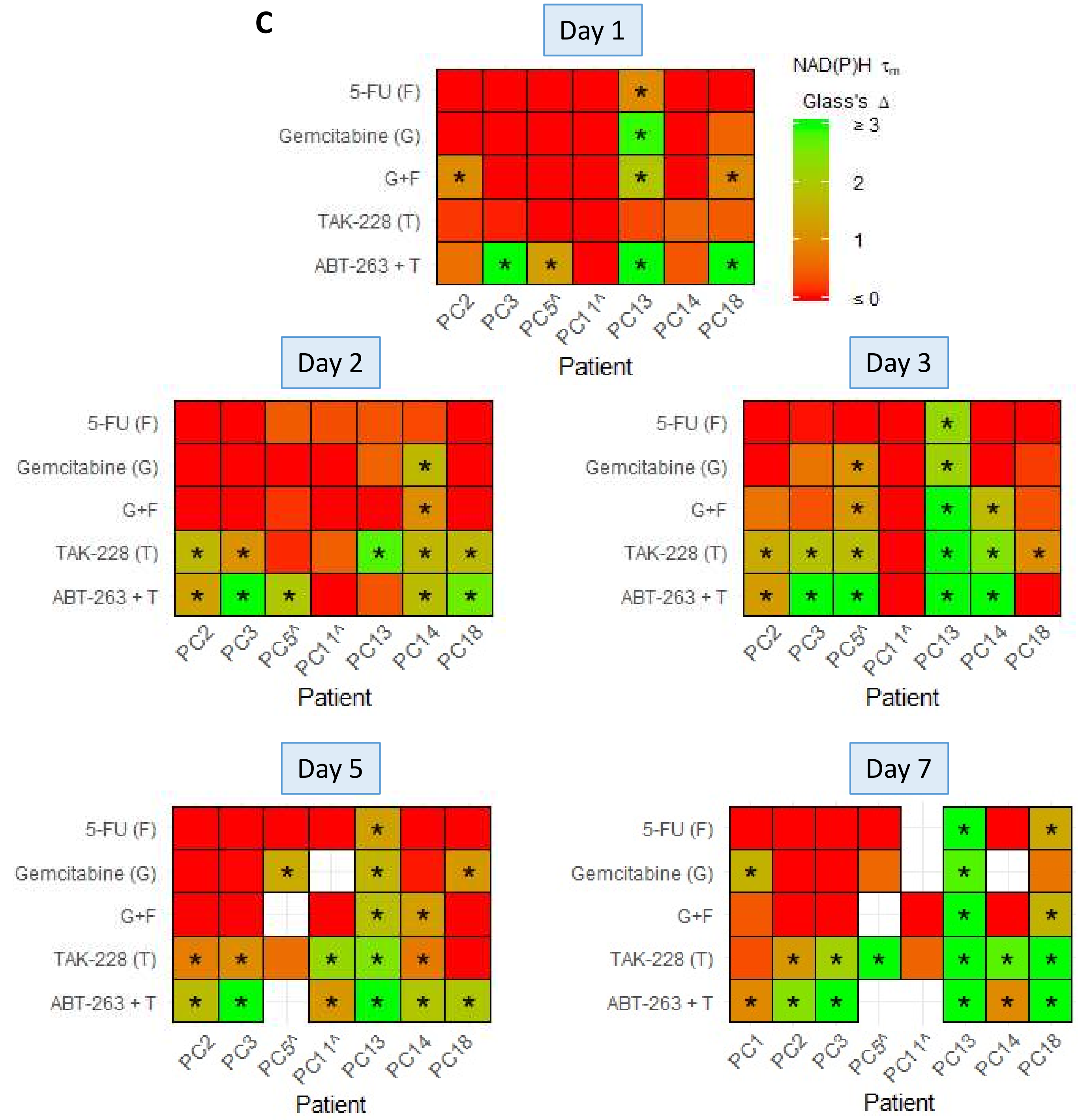
C
Day 1
NAD(P)H τ_m
Glass's Δ
≥ 3
2
1
≤ 0
5-FU (F)
Gemcitabine (G)
G+F
TAK-228 (T)
ABT-263 + T
PC2
PC3
PC5^
PC11^
PC13
PC14
PC18
Patient
Day 2
Day 3
Day 5
Day 7
PC1
*

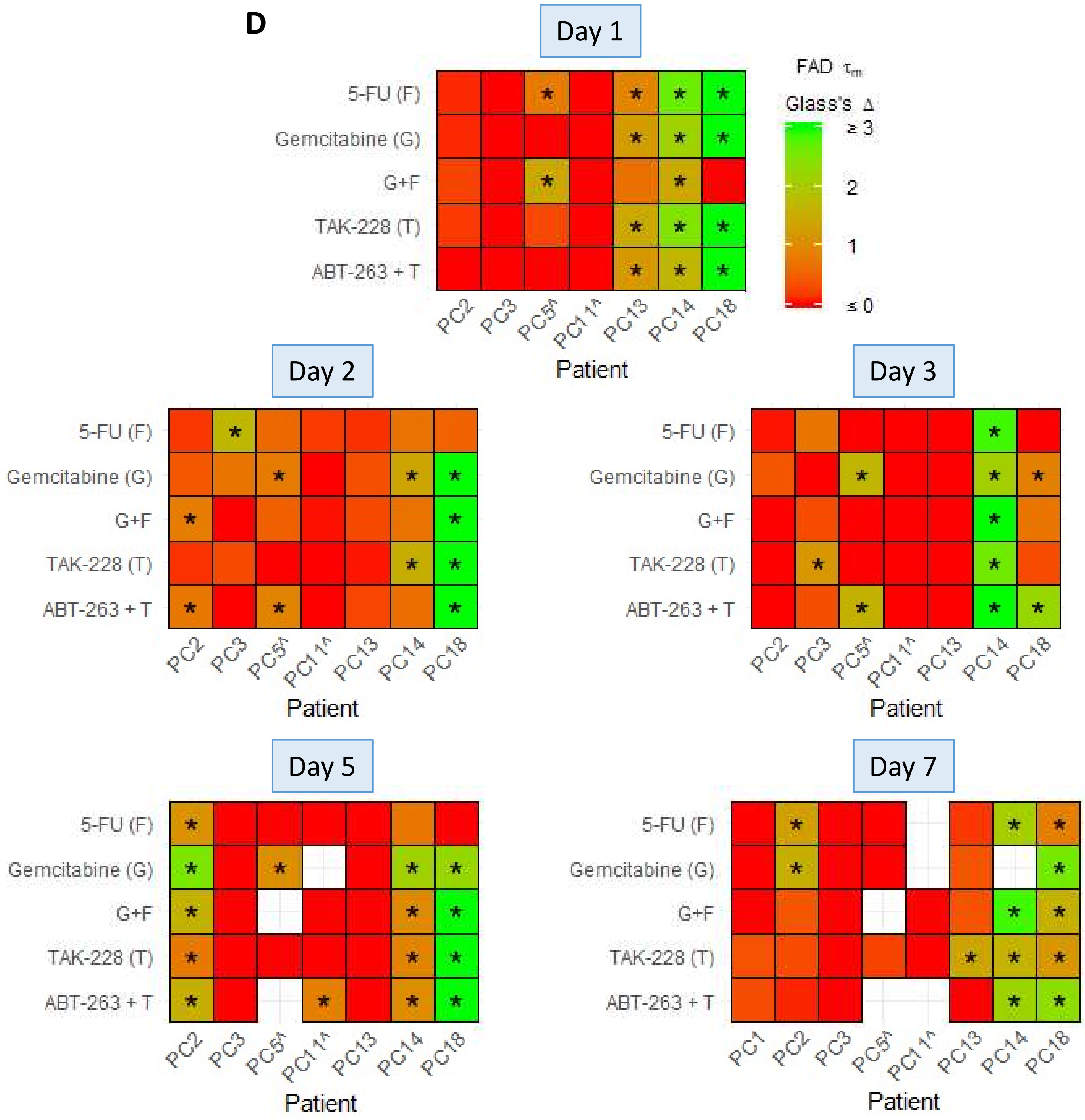

Supplementary Figure S5. Effect sizes of drug treatment on individual OMI endpoints in pancreas patient-derived fibroblasts co-cultured with organoids. A-D, Heatmap representation of the treatment effect size (Glass's Δ) at each time point for the OMI index (**A**), redox ratio (**B**), NAD(P)H τ_m (**C**), and FAD τ_m (**D**). '^' indicates the patient lesion was diagnosed as PanIN. '~' indicates the patient lesion was diagnosed as ampullary cancer. * Glass's $\Delta \geq 0.75$ vs. control.

A

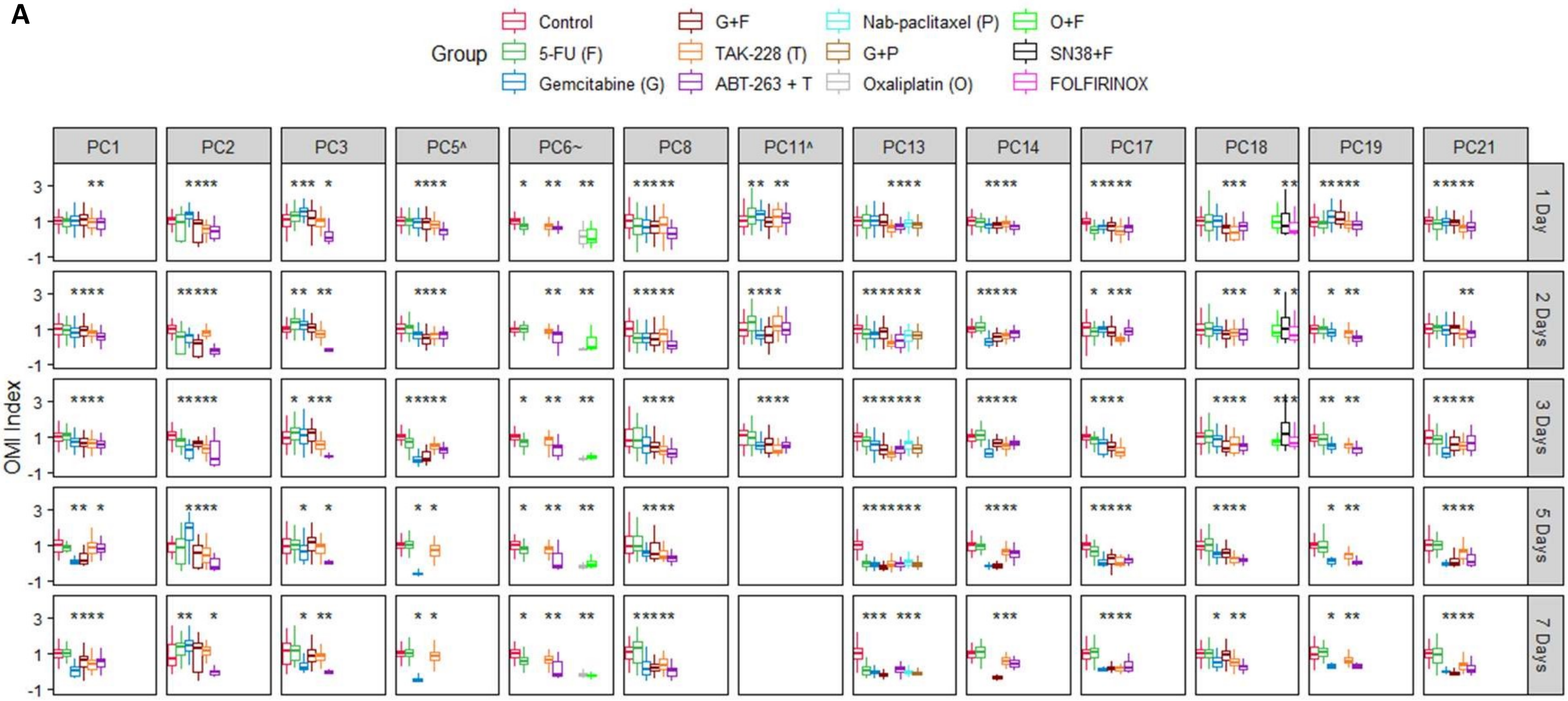
Group
Control
5-FU (F)
Gemcitabine (G)
G+F
TAK-228 (T)
ABT-263 + T
Nab-paclitaxel (P)
G+P
Oxaliplatin (O)
O+F
SN38+F
FOLFIRINOX
PC1
PC2
PC3
PC5^
PC6~
PC8
PC11^
PC13
PC14
PC17
PC18
PC19
PC21
1 Day
2 Days
3 Days
5 Days
7 Days
OMI Index

B

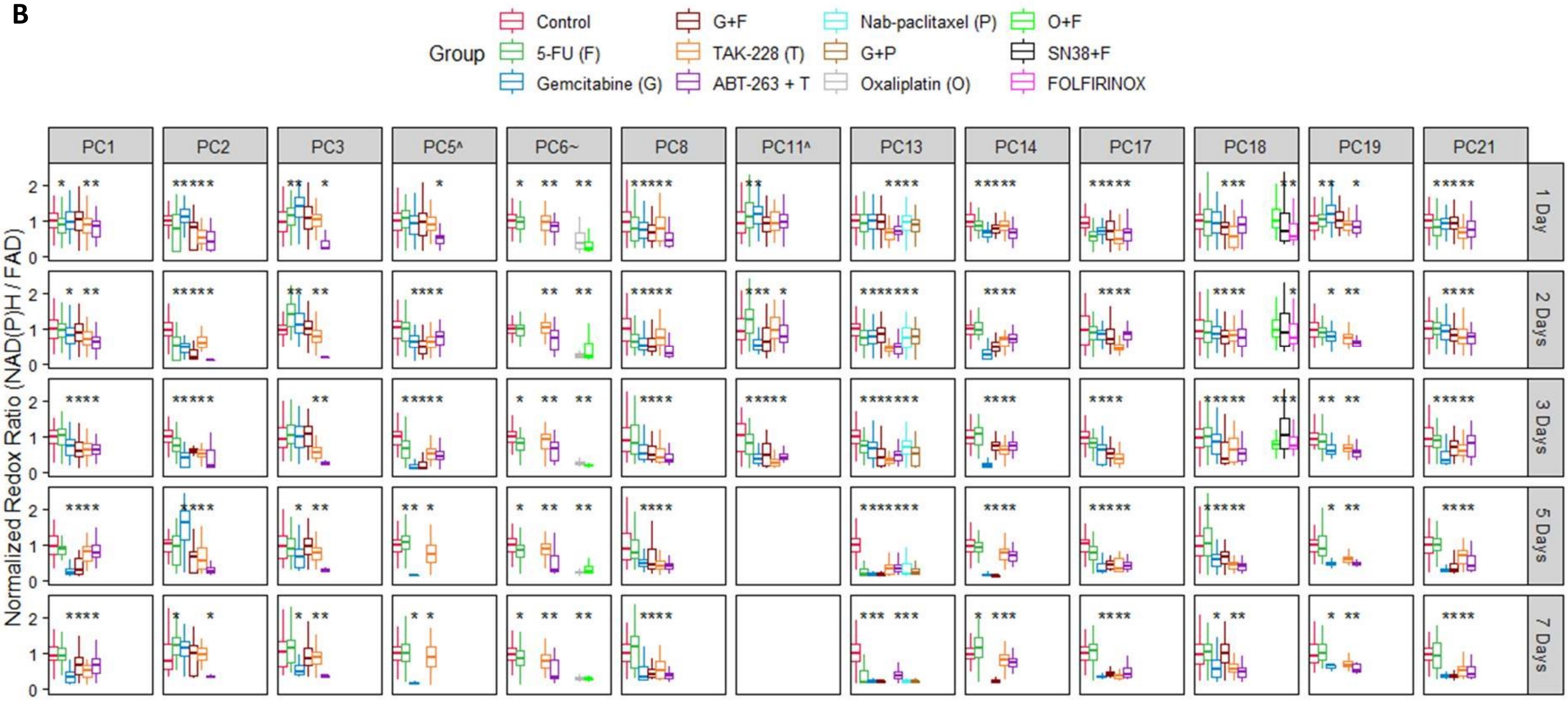
Group
Control
5-FU (F)
Gemcitabine (G)
G+F
TAK-228 (T)
ABT-263 + T
Nab-paclitaxel (P)
G+P
Oxaliplatin (O)
O+F
SN38+F
FOLFIRINOX
PC1
PC2
PC3
PC5^
PC6~
PC8
PC11^
PC13
PC14
PC17
PC18
PC19
PC21
1 Day
2 Days
3 Days
5 Days
7 Days
Normalized Redox Ratio (NAD(P)H / FAD)

C

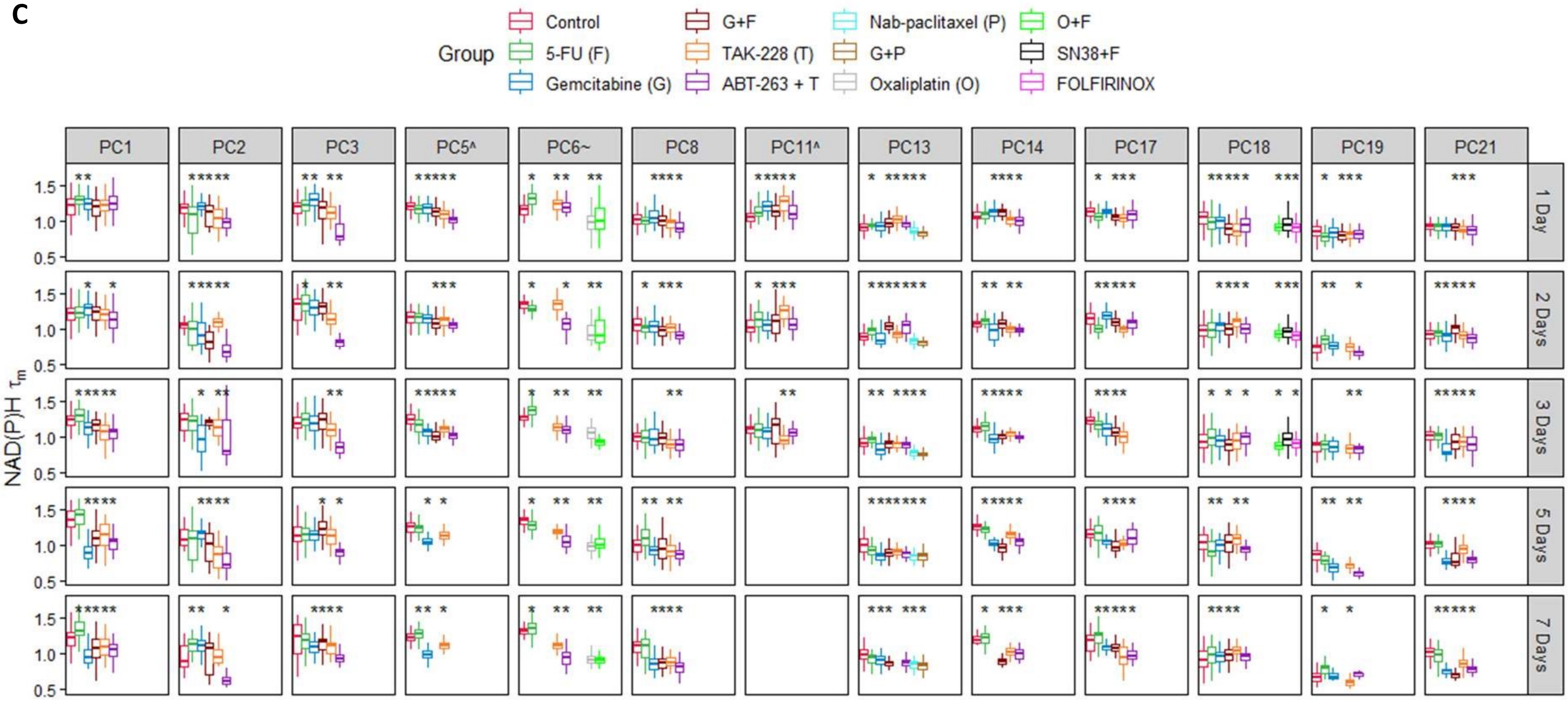
Group
Control
5-FU (F)
Gemcitabine (G)
G+F
TAK-228 (T)
ABT-263 + T
Nab-paclitaxel (P)
G+P
Oxaliplatin (O)
O+F
SN38+F
FOLFIRINOX
PC1
PC2
PC3
PC5^
PC6~
PC8
PC11^
PC13
PC14
PC17
PC18
PC19
PC21
1 Day
2 Days
3 Days
5 Days
7 Days
NAD(P)H τ_m

D

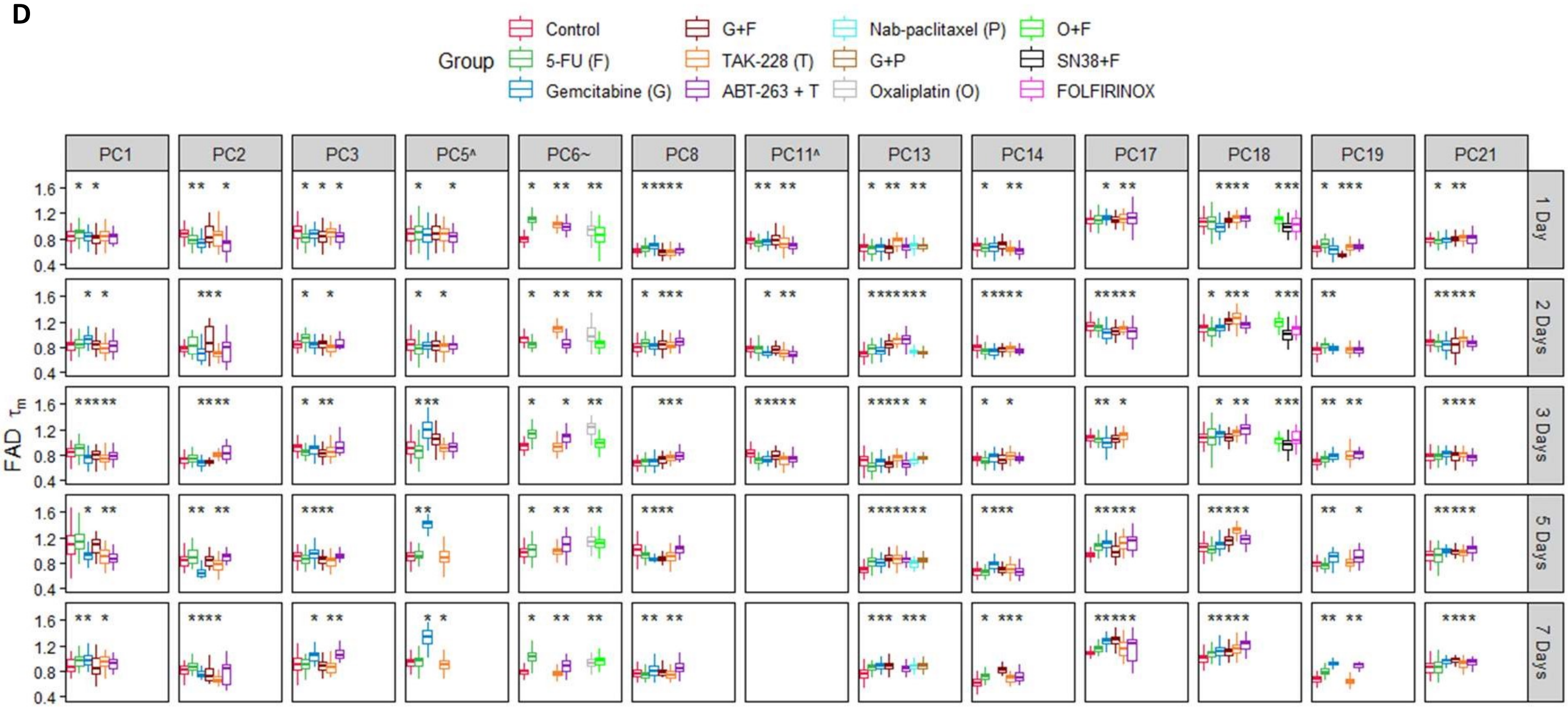
Group
Control
5-FU (F)
Gemcitabine (G)
G+F
TAK-228 (T)
ABT-263 + T
Nab-paclitaxel (P)
G+P
Oxaliplatin (O)
O+F
SN38+F
FOLFIRINOX
PC1
PC2
PC3
PC5^
PC6~
PC8
PC11^
PC13
PC14
PC17
PC18
PC19
PC21
1 Day
2 Days
3 Days
5 Days
7 Days
FAD τ_m

Supplementary Figure S6. Significance of drug treatment effects on individual OMI endpoints in pancreatic patient-derived organoids. A-D, Boxplot summaries comparing the effect of all drugs between patients and time points in organoids on the OMI index (**A**), redox ratio (**B**), NAD(P)H τ_m **(C)**, and FAD τ_m (**D**). '^' indicates the patient lesion was diagnosed as PanIN. '~' indicates the patient lesion was diagnosed as ampullary cancer. * p<0.05 vs. control.

A

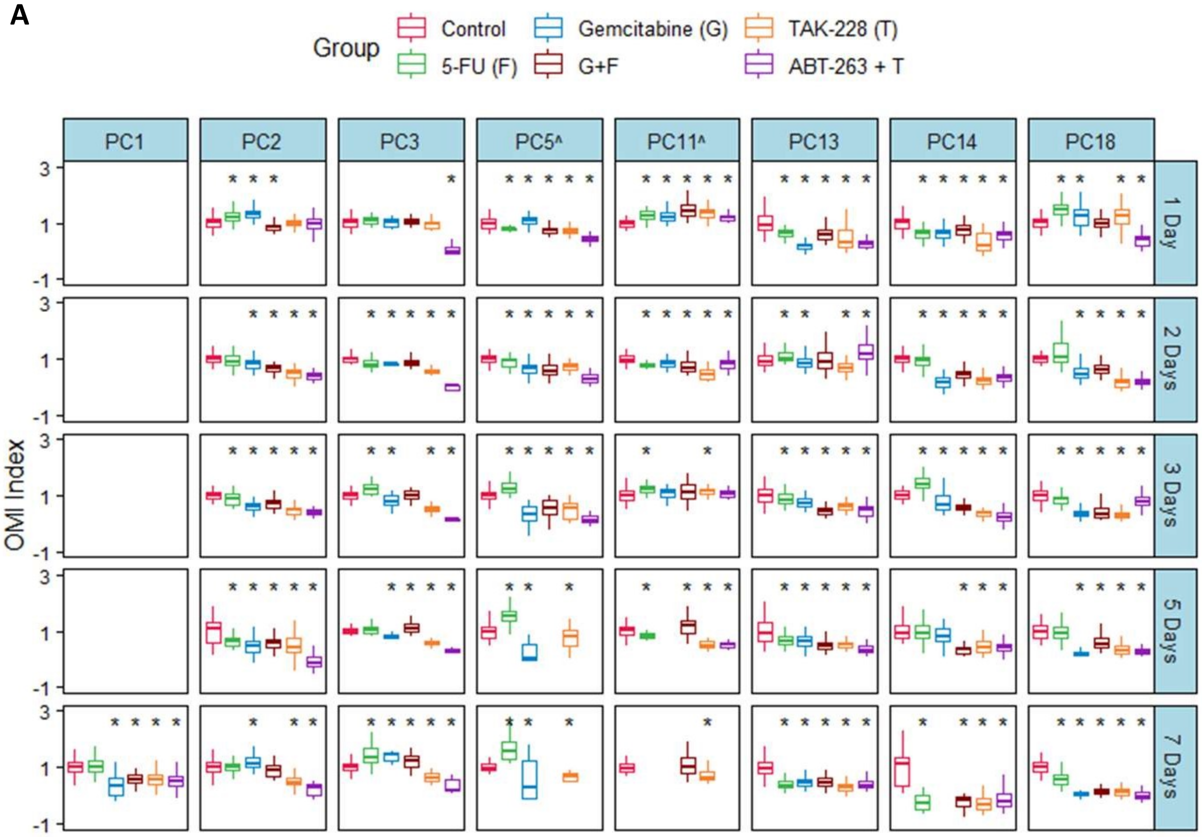
Group
Control
Gemcitabine (G)
TAK-228 (T)
5-FU (F)
G+F
ABT-263 + T
PC1
PC2
PC3
PC5^
PC11^
PC13
PC14
PC18
1 Day
2 Days
3 Days
5 Days
7 Days
OMI Index
3
1
-1

B

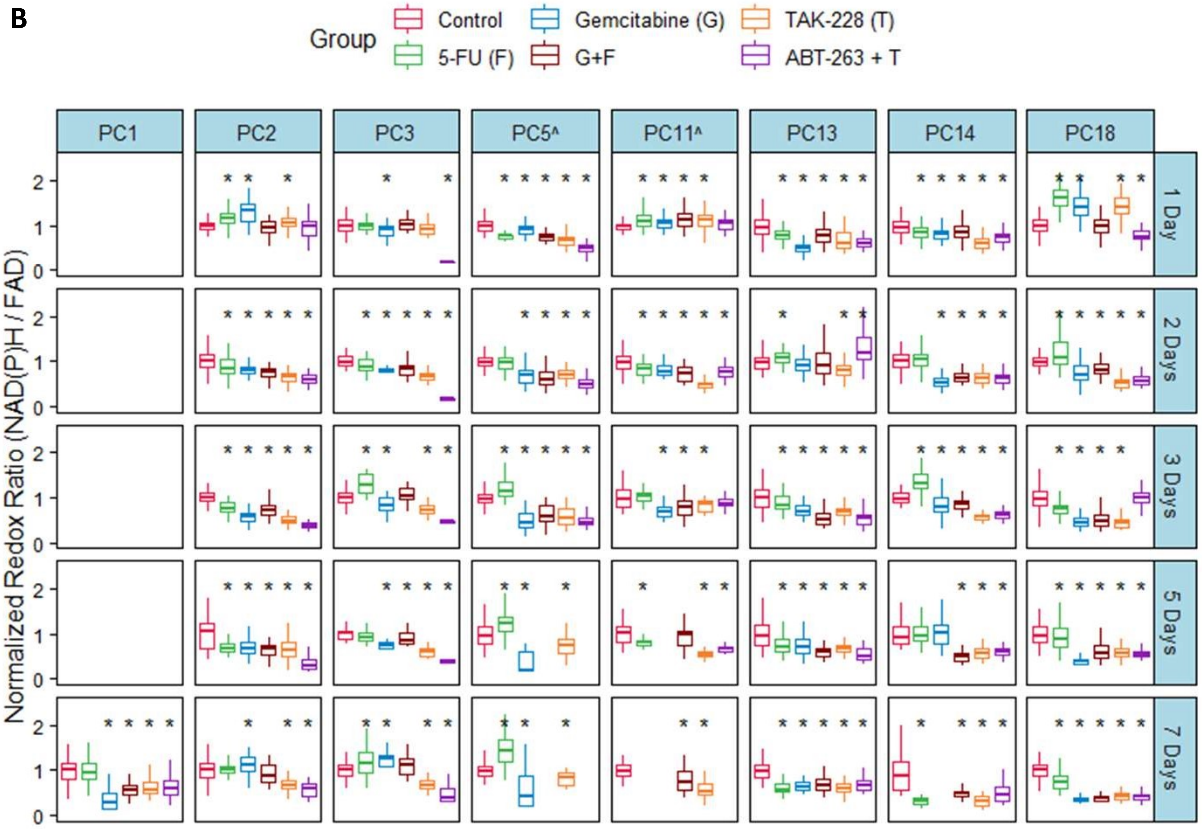
Group
Control
Gemcitabine (G)
TAK-228 (T)
5-FU (F)
G+F
ABT-263 + T
PC1
PC2
PC3
PC5^
PC11^
PC13
PC14
PC18
1 Day
2 Days
3 Days
5 Days
7 Days
Normalized Redox Ratio (NAD(P)H / FAD)
2
1
0

C

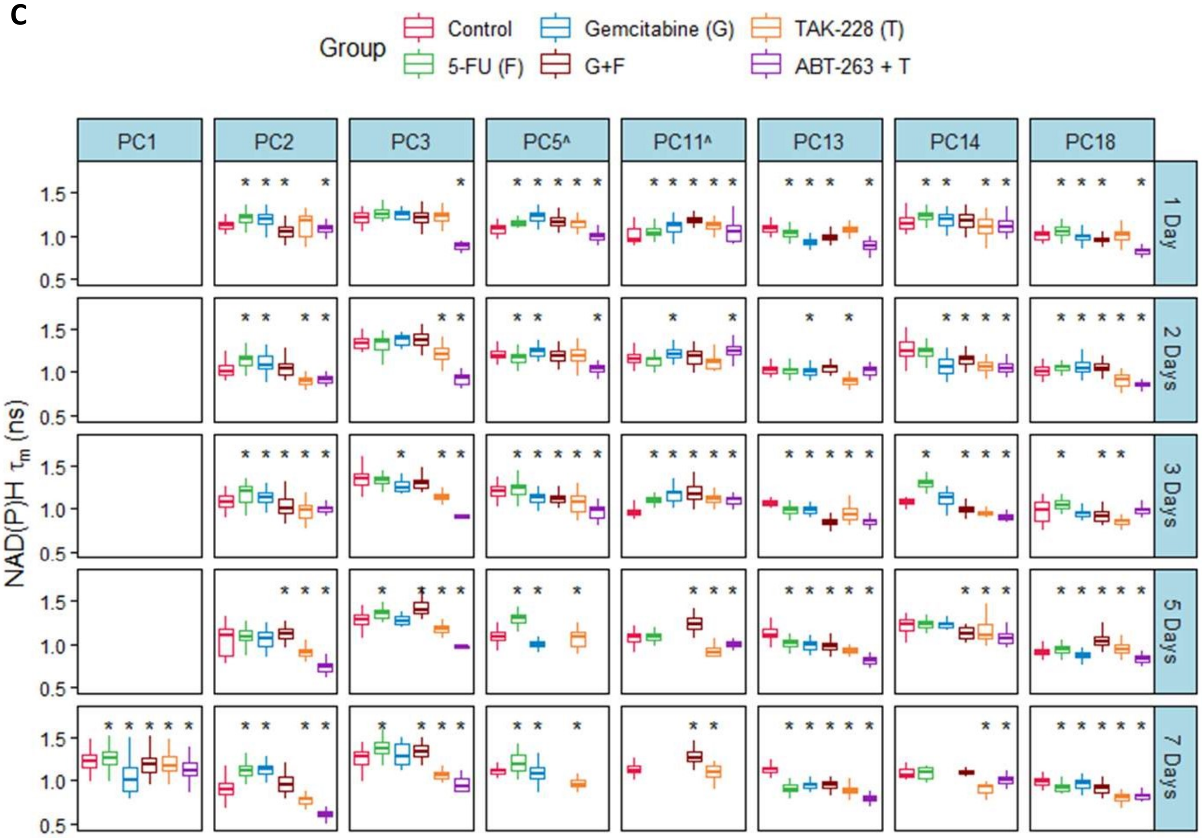

Group
Control
5-FU (F)
Gemcitabine (G)
G+F
TAK-228 (T)
ABT-263 + T
PC1
PC2
PC3
PC5^
PC11^
PC13
PC14
PC18
1 Day
2 Days
3 Days
5 Days
7 Days
NAD(P)H τm (ns)

D

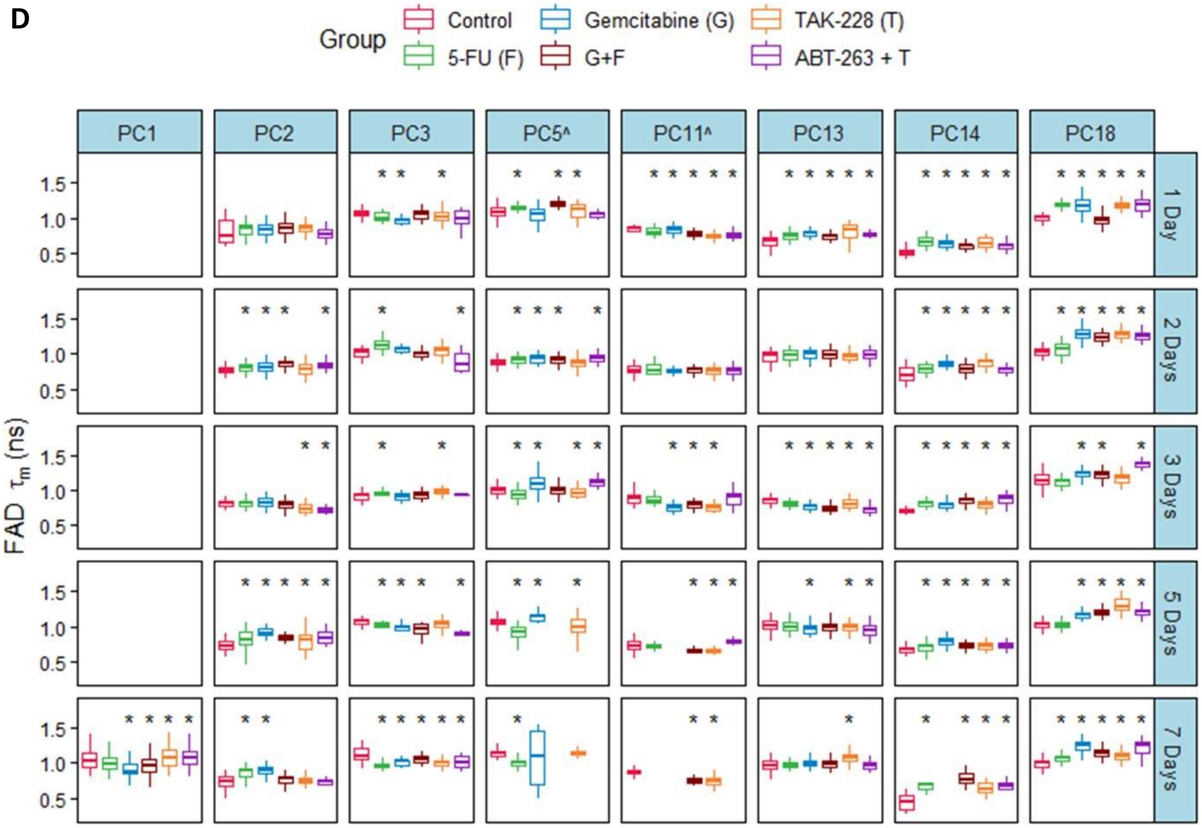

Group
Control
5-FU (F)
Gemcitabine (G)
G+F
TAK-228 (T)
ABT-263 + T
PC1
PC2
PC3
PC5^
PC11^
PC13
PC14
PC18
1 Day
2 Days
3 Days
5 Days
7 Days
FAD τm (ns)

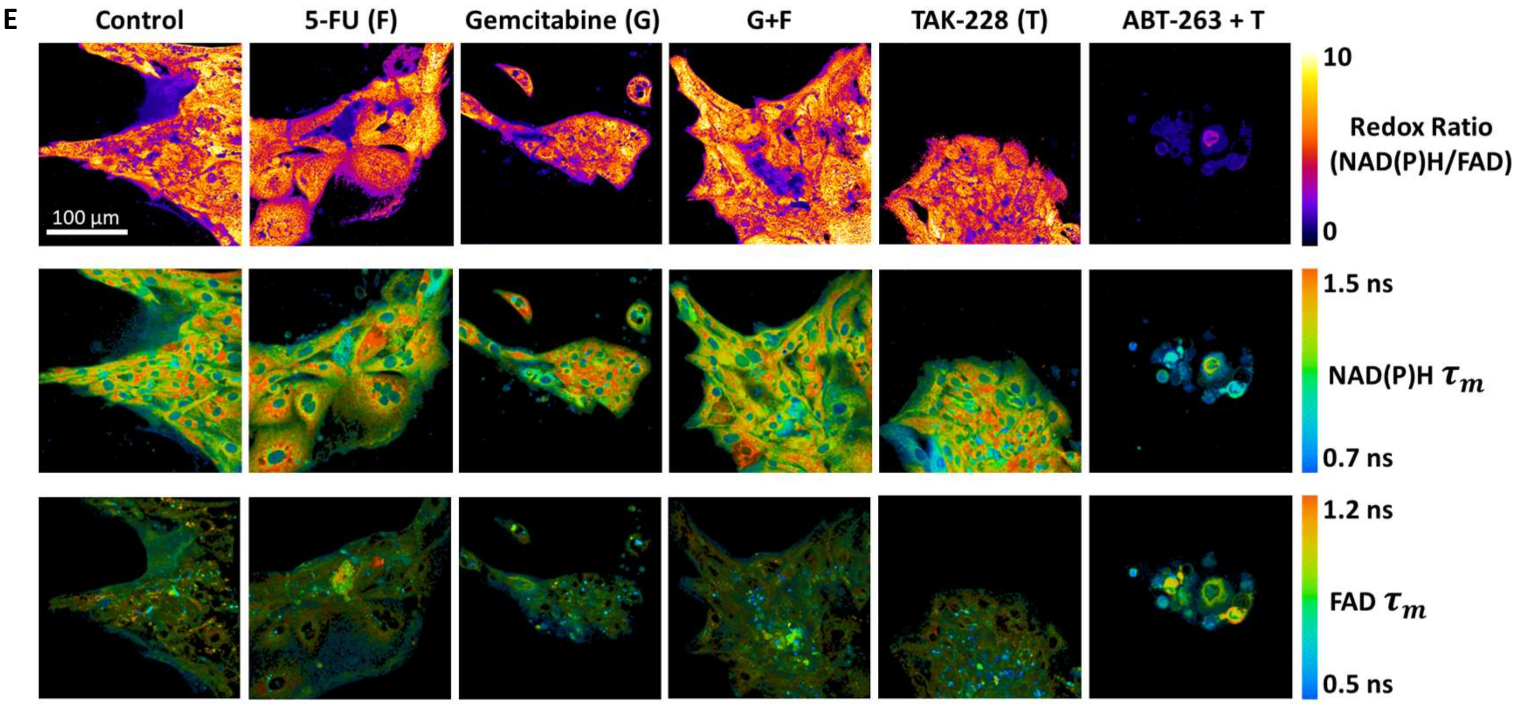

Supplementary Figure S7. Significance of drug treatment effects on individual OMI endpoints in pancreas patient-derived fibroblasts co-cultured with organoids. A-D, Boxplot summaries comparing the effect of all drugs on patient-derived fibroblasts at all time points for the OMI index (**A**), redox ratio (**B**), NAD(P)H τ_m (**C**), and FAD τ_m (**D**). '^' indicates the patient lesion was diagnosed as PanIN. '~' indicates the patient lesion was diagnosed as ampullary cancer. * p<0.05 vs. control. **E,** Representative OMI images of fibroblast monolayer co-cultured with organoids derived from Patient PC3 at 24 hours of treatment.

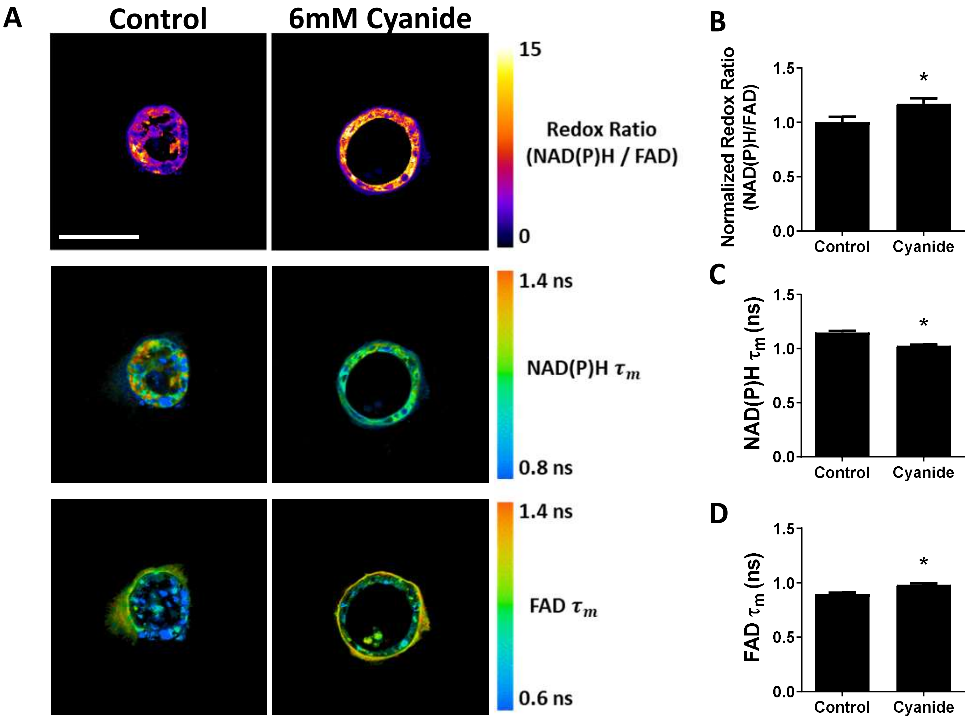

Supplementary Figure S8. Metabolic perturbation with cyanide validates OMI and organoid viability. A, Representative redox ratio, NAD(P)H τ_m, and FAD τ_m images of Patient PC1 organoids before and after 6mM cyanide treatment. Scale bar is 100 µm. **B,** Optical redox ratio increases with cyanide. **C,** NAD(P)H mean lifetime decreases with cyanide. **D,** FAD mean lifetime increases with cyanide treatment. Error bars indicate mean ± SD. * $p<0.05$. N=4 pancreatic organoids comprising 64 cells.

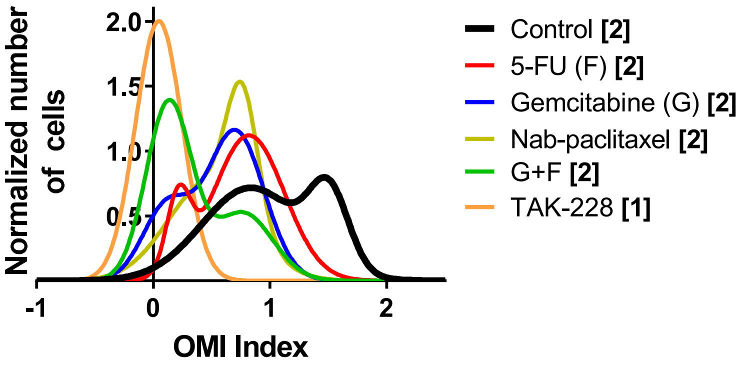

Supplementary Figure S9. Population distribution modeling in response to additional drugs in Patient PC13 organoids. Normalized density distributions of the OMI index of individual cells after 72 hours. Bracketed number indicates number of subpopulations.

A

B

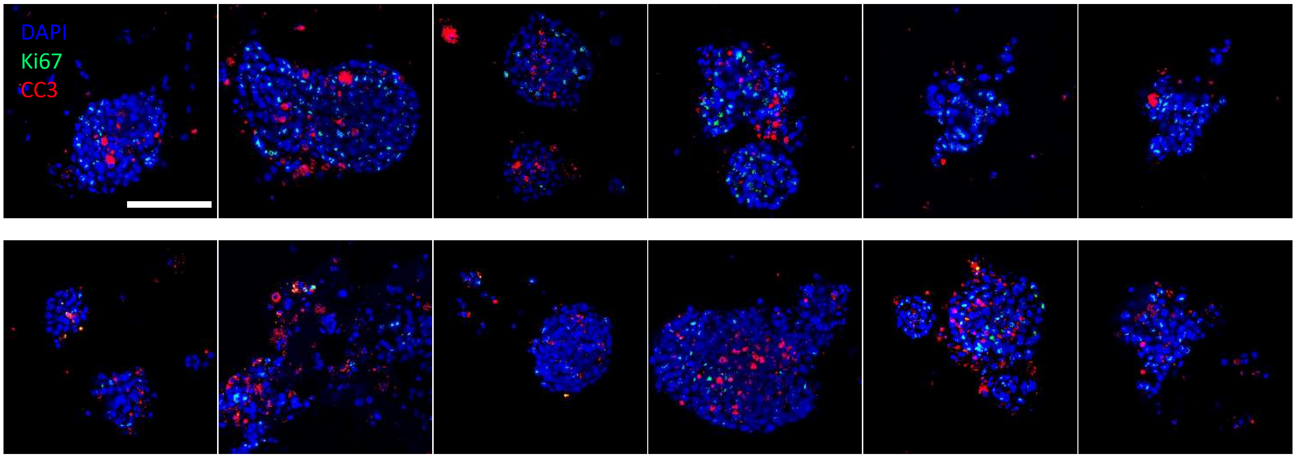

Supplementary Figure S10. Dual immunofluorescence of cell fate in pancreatic organoids. Composite images of PC13 organoids stained for Ki67 (green), cleaved caspase-3 (red), and DAPI (blue). **A,** Control organoids. Scale bar is 100 μm. **B,** organoids treated for 72 hours with the combination of TAK-228 and ABT-263.

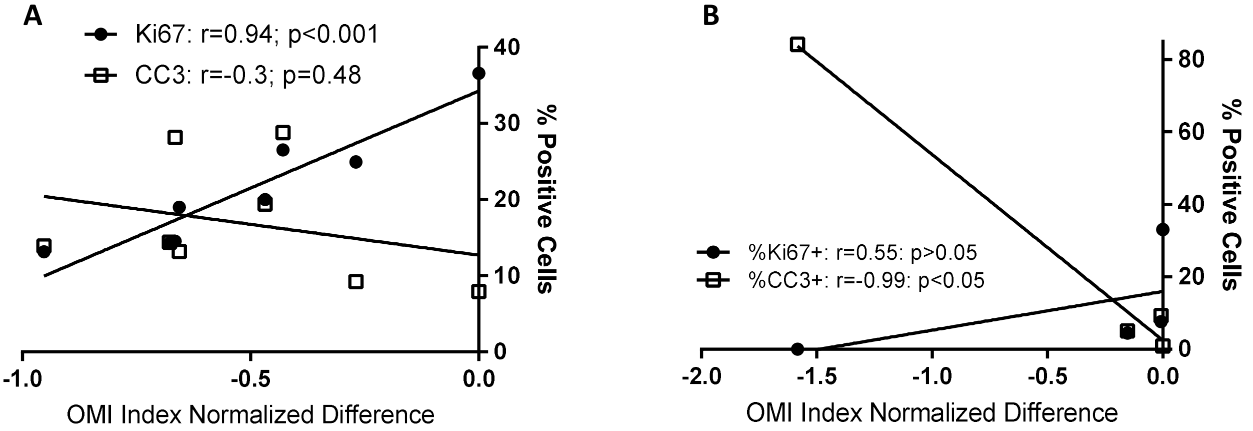

Supplementary Figure S11. Correlation between OMI index, proliferation, and apoptosis in human organoids. **A,** In PC13 patient organoids, OMI index positively correlates with Ki67 expression (filled circles). OMI index does not significantly correlate with CC3 expression (open squares). Each dot represents the average value for one treatment group at 72 hours of treatment. N=8 conditions. **B,** In BC8 patient organoids, OMI index does not significantly correlate with percentage of cells expressing Ki67, but does significantly correlate with percentage of cells expressing CC3. Each dot represents the average value for one treatment group at 72 hours of treatment. N=4 conditions.

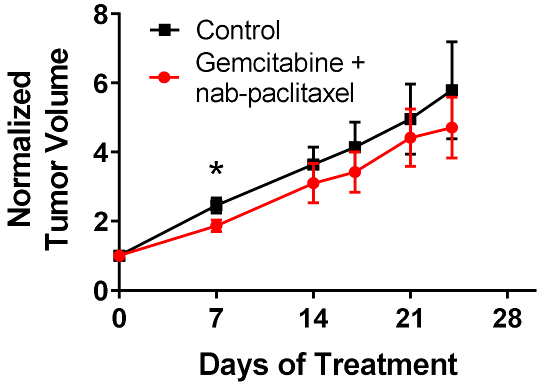

Supplementary Figure S12. Growth response to gemcitabine and nab-paclitaxel combination therapy in patient-derived xenograft tumors grown from Patient PC13 organoids. Error bars indicate mean ± SEM. * $p<0.05$ vs. control. N>20 tumors per group at day 7, N>13 tumors per group at day 14 and beyond.

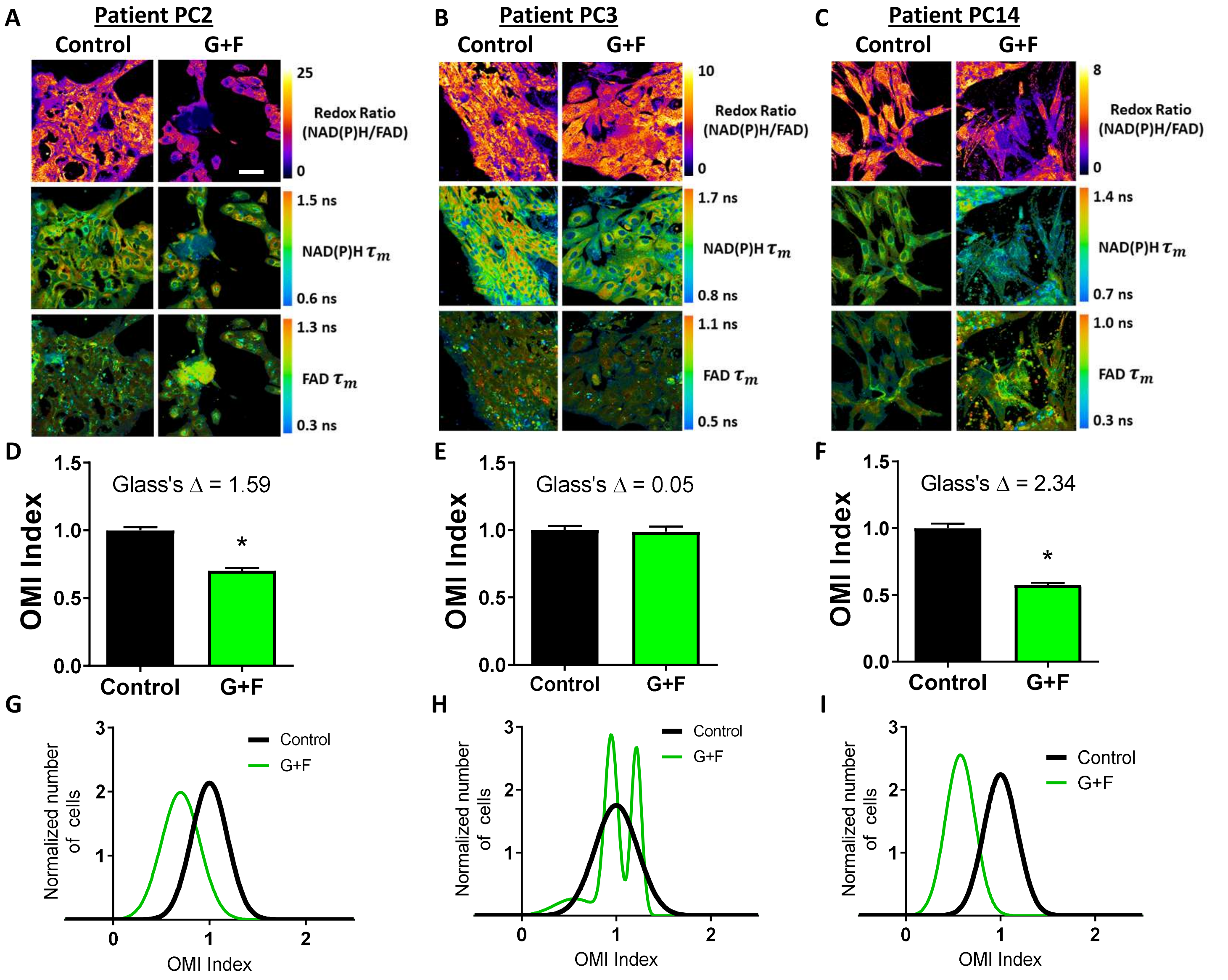

Supplementary Figure S13. Response to patient adjuvant therapy in patient-derived fibroblasts. A-C, Representative redox ratio, NAD(P)H τ_m, and FAD τ_m images of fibroblasts co-cultured with organoids from Patients PC2 (**A**), PC3 (**B**), and PC14 (**C**). Left columns indicate control fibroblasts, and right columns indicate fibroblasts treated with drugs matched to the patient's adjuvant treatment. G+F = gemcitabine + 5-FU. Scale bar is 50 µm. **D-F,** The effect of the same drugs on the OMI index averaged across all fibroblasts derived from Patient PC2 (**D**), PC3 (**E**), and PC14 (**F**). Error bars indicate mean ± SEM. * p<0.0001. **G-I,** Single-cell OMI index subpopulation analysis of treatment response in fibroblasts from Patient PC2 (**G**), PC3 (**H**), and PC14 (**I**).

A

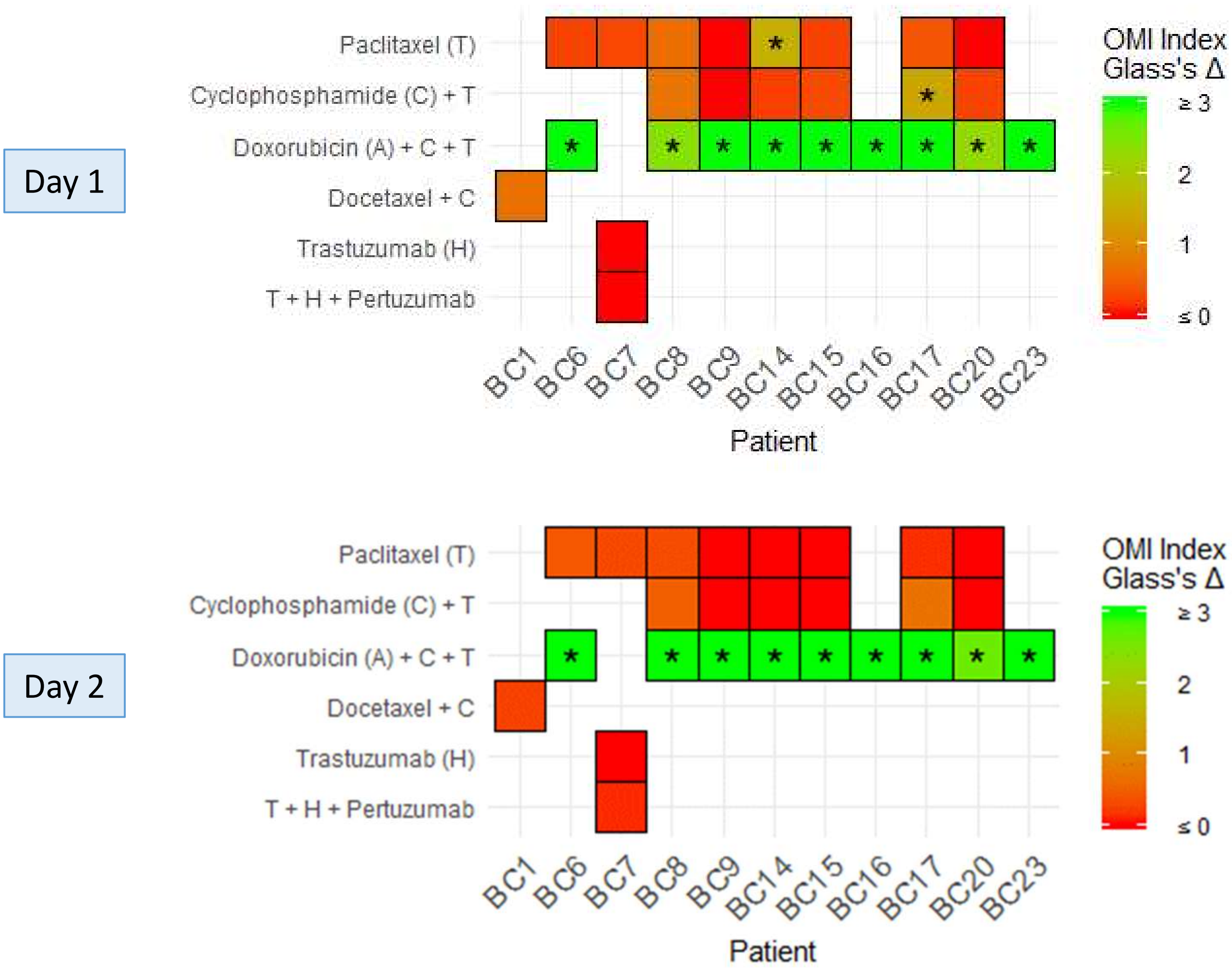
Day 1
Paclitaxel (T)
Cyclophosphamide (C) + T
Doxorubicin (A) + C + T
Docetaxel + C
Trastuzumab (H)
T + H + Pertuzumab
BC1 BC6 BC7 BC8 BC9 BC14 BC15 BC16 BC17 BC20 BC23
Patient
OMI Index
Glass's Δ
≥ 3
2
1
≤ 0
Day 2
Paclitaxel (T)
Cyclophosphamide (C) + T
Doxorubicin (A) + C + T
Docetaxel + C
Trastuzumab (H)
T + H + Pertuzumab
BC1 BC6 BC7 BC8 BC9 BC14 BC15 BC16 BC17 BC20 BC23
Patient
OMI Index
Glass's Δ
≥ 3
2
1
≤ 0

B

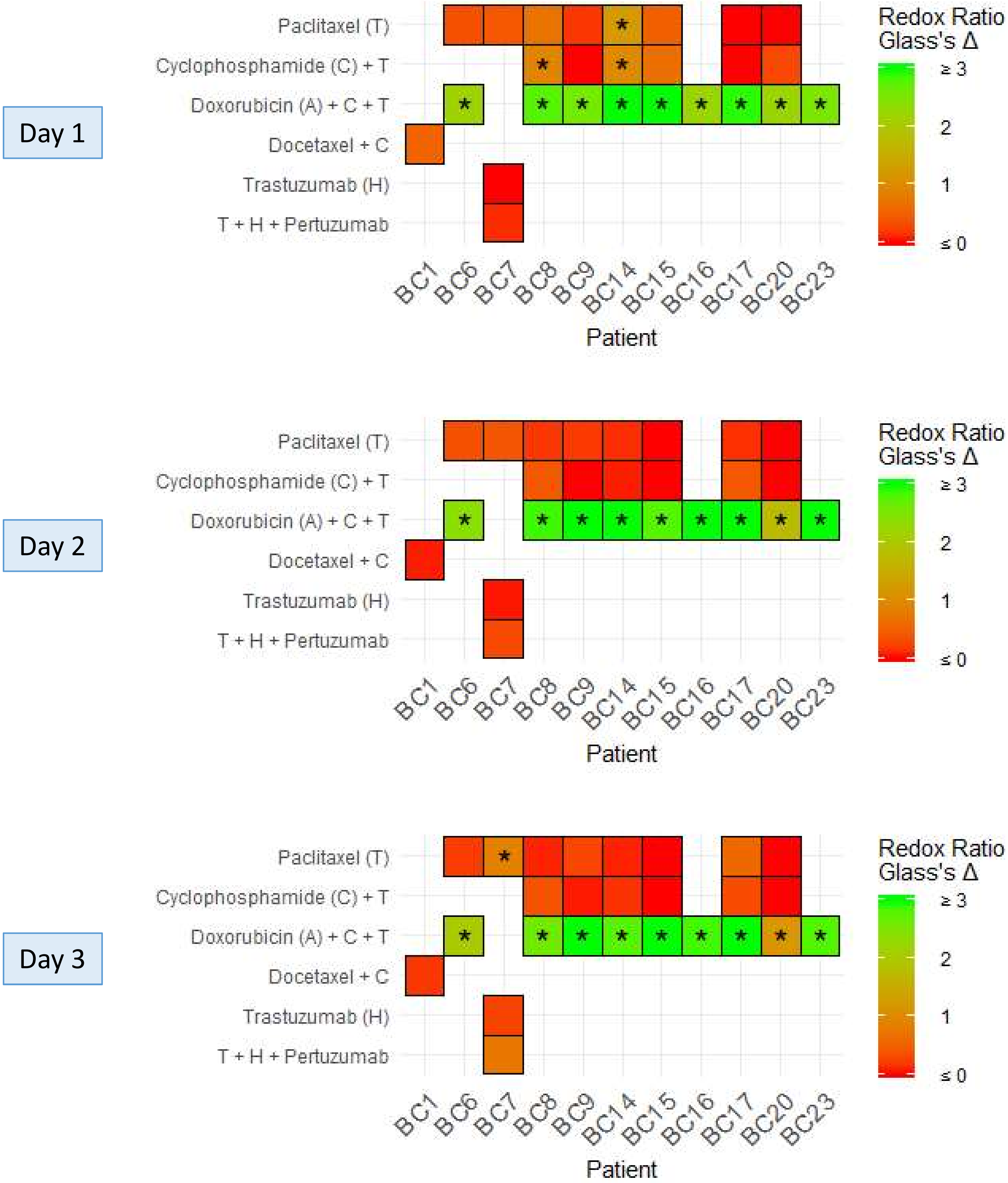
Day 1
Day 2
Day 3
Paclitaxel (T)
Cyclophosphamide (C) + T
Doxorubicin (A) + C + T
Docetaxel + C
Trastuzumab (H)
T + H + Pertuzumab
BC1
BC6
BC7
BC8
BC9
BC14
BC15
BC16
BC17
BC20
BC23
Patient
Redox Ratio
Glass's Δ
≥ 3
2
1
≤ 0
*

C

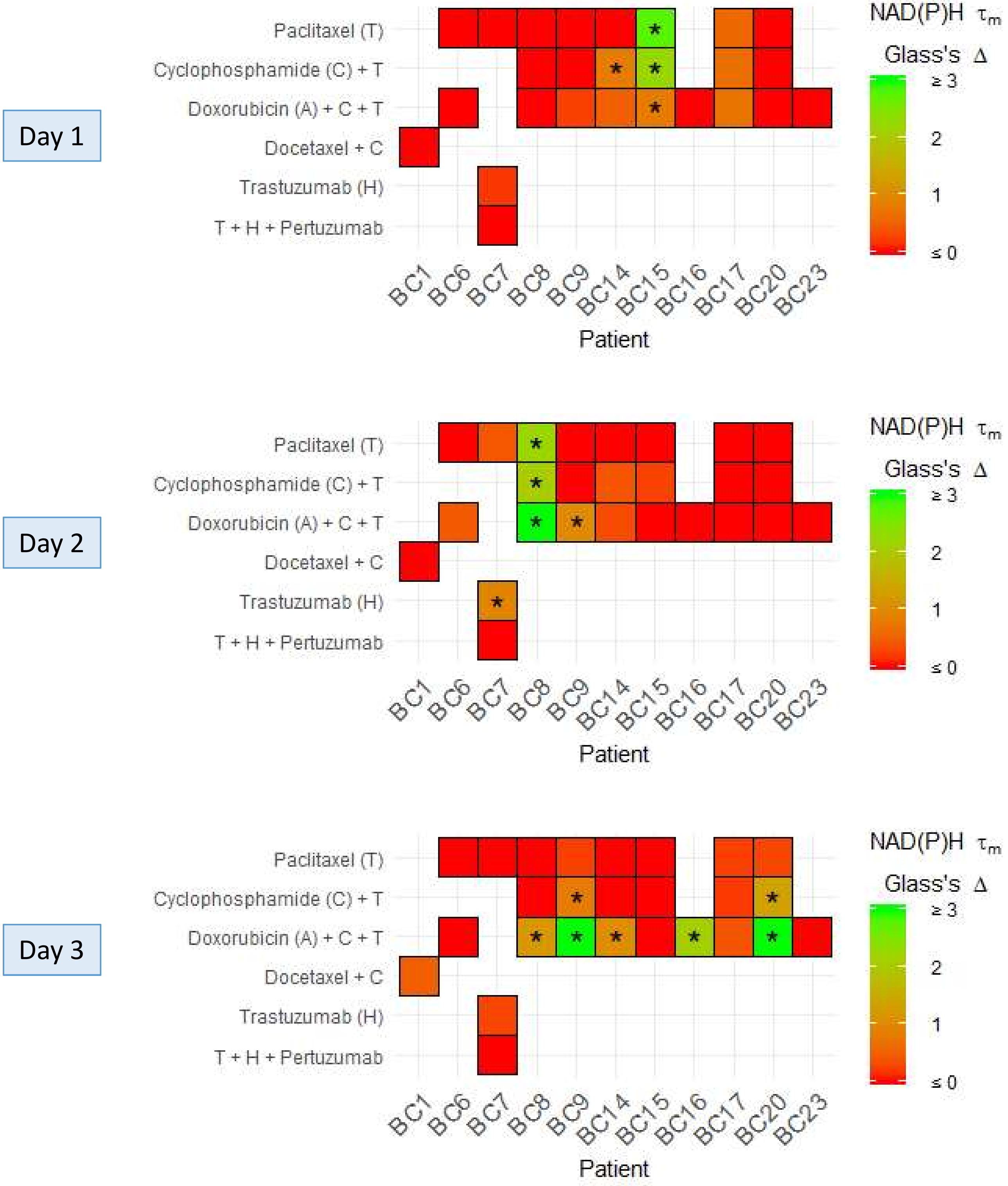

Day 1
Day 2
Day 3
Paclitaxel (T)
Cyclophosphamide (C) + T
Doxorubicin (A) + C + T
Docetaxel + C
Trastuzumab (H)
T + H + Pertuzumab
BC1
BC6
BC7
BC8
BC9
BC14
BC15
BC16
BC17
BC20
BC23
Patient
NAD(P)H τm
Glass's Δ
≥ 3
2
1
≤ 0
*

D

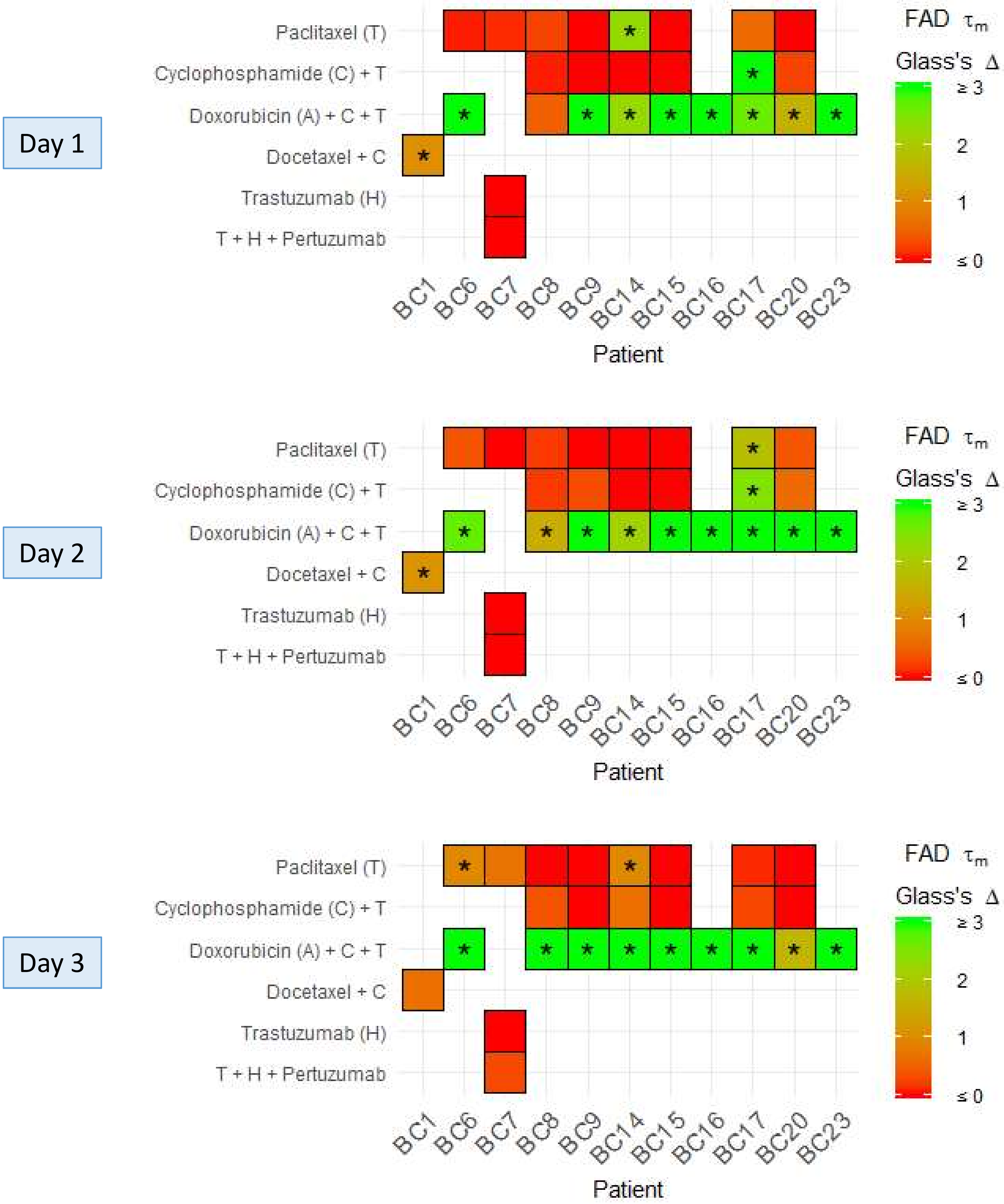

Day 1
Day 2
Day 3
Paclitaxel (T)
Cyclophosphamide (C) + T
Doxorubicin (A) + C + T
Docetaxel + C
Trastuzumab (H)
T + H + Pertuzumab
BC1
BC6
BC7
BC8
BC9
BC14
BC15
BC16
BC17
BC20
BC23
Patient
FAD τ_m
Glass's Δ
≥ 3
2
1
≤ 0

Supplementary Figure S14. Effect sizes of drug treatment on individual OMI endpoints in patient-derived breast cancer organoids. A-D, Heatmap representation of the treatment effect size (Glass's Δ) at each time point for the OMI index (**A**), redox ratio (**B**), NAD(P)H τ_m (**C**), and FAD τ_m (**D**). * Glass's $\Delta \geq 0.75$ vs. control. 4-OOH cyclophosphamide (active metabolite) was used in place of cyclophosphamide.

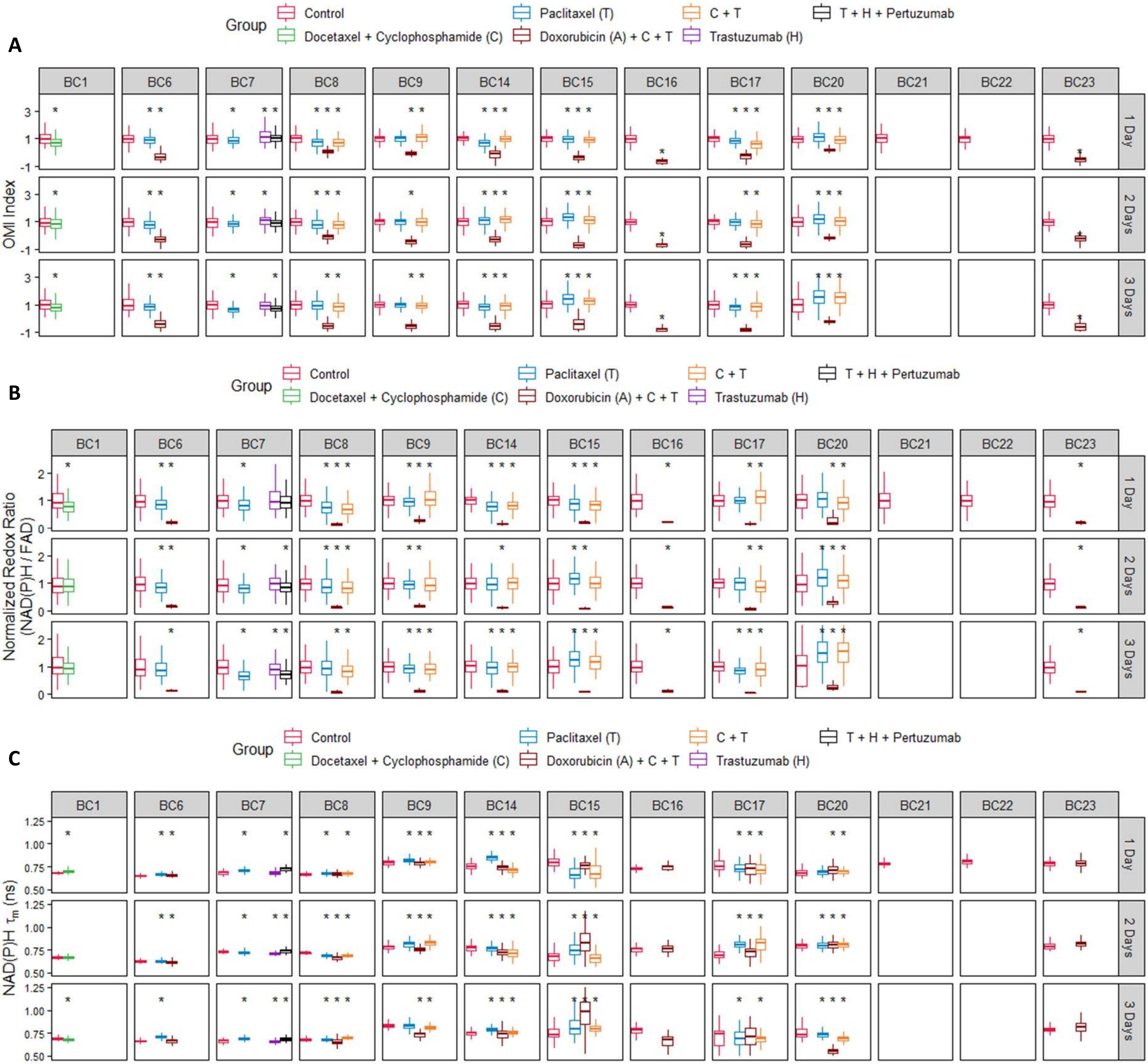
A
B
C
Group
Control
Docetaxel + Cyclophosphamide (C)
Paclitaxel (T)
Doxorubicin (A) + C + T
C + T
Trastuzumab (H)
T + H + Pertuzumab
BC1
BC6
BC7
BC8
BC9
BC14
BC15
BC16
BC17
BC20
BC21
BC22
BC23
1 Day
2 Days
3 Days
OMI Index
Normalized Redox Ratio
(NAD(P)H / FAD)
NAD(P)H τ_m (ns)

D

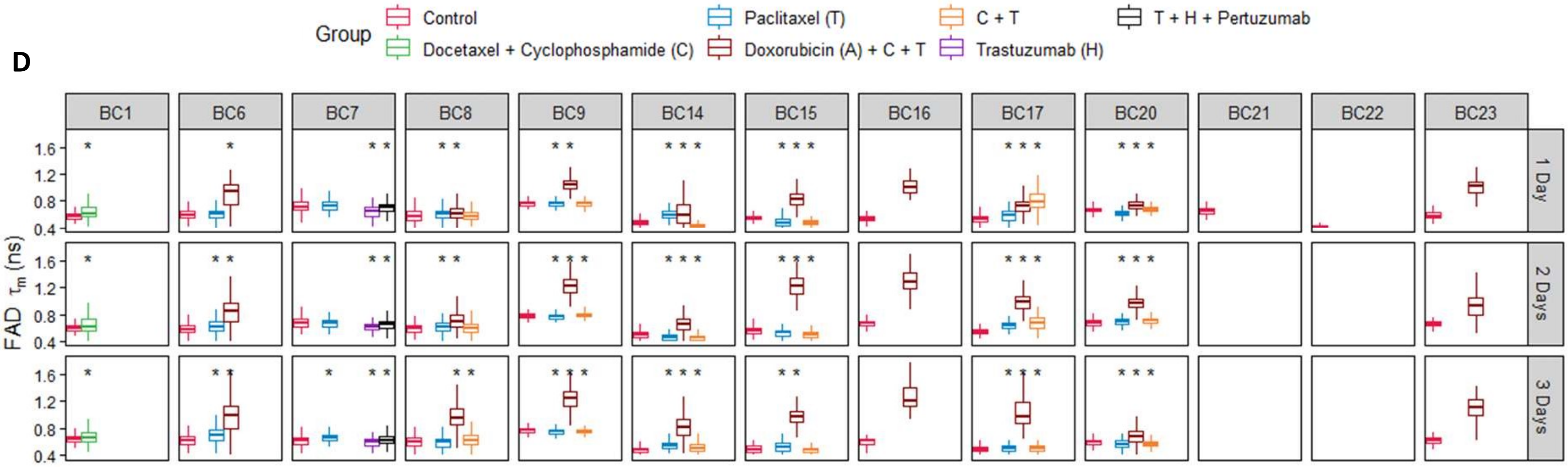

Supplementary Figure S15. Significance of drug treatment effects on individual OMI endpoints in breast cancer patient-derived organoids. A-D, Boxplot summaries comparing the effect of all drugs between patients and time points in organoids on the OMI index (**A**), redox ratio (**B**), NAD(P)H τ_m **(C)**, and FAD τ_m (**D**). * p<0.05 vs. control. 4-OOH cyclophosphamide (active metabolite) was used in place of cyclophosphamide.

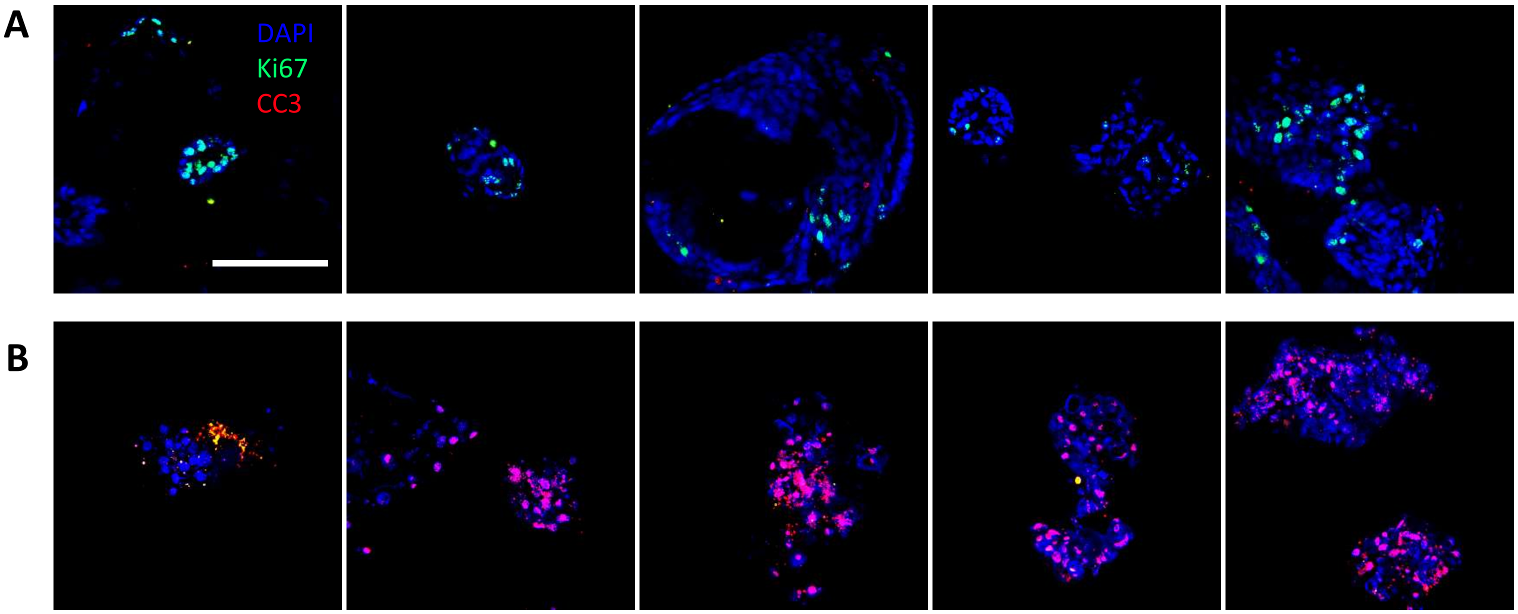

Supplementary Figure S16. Dual immunofluorescence of cell fate in breast cancer organoids. Composite images of BC8 organoids stained for Ki67 (green), cleaved caspase-3 (red), and DAPI (blue). **A,** Control organoids. Scale bar is 100 µm. **B,** organoids treated for 72 hours with A+C+T combination chemotherapy.

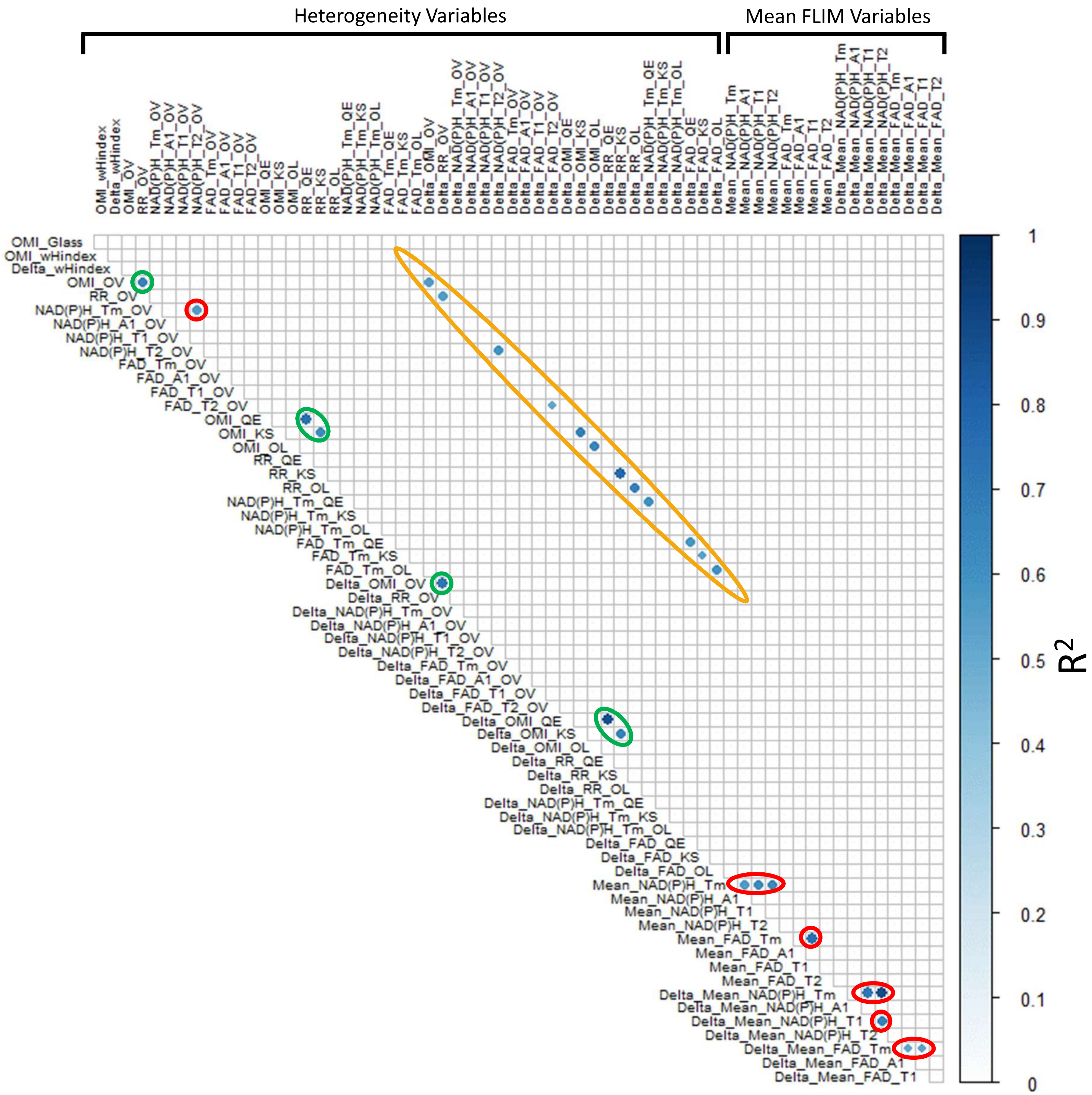

Heterogeneity Variables
Mean FLIM Variables
R2

Supplementary Figure S17. Bivariate correlations between changes in heterogeneity and mean FLIM variables with treatment. R^2 strengths of correlations between all post-treatment heterogeneity measurements and mean metabolic measurements, as well as all changes in these variables with treatment. Includes all BC and PC patients, treatments, and time points (N=401 total treatment/control pairs). OV = percentage of cellular variation explained by organoid variation, KS = Kolmogorov-Smirnov distance, QE = quadratic entropy, OL = outlier percentage, OMI = OMI index, RR = redox ratio, Glass = Glass's Δ effect size. Differences between pre- and post-treatment variables have "Delta_" prefix. Correlations with $R^2 < 0.5$ are omitted for clarity. Red highlights include correlations between τ_m, τ_1, τ_2, α_1 of one FLIM measurement. Green highlights include correlations between OMI index variables and paired redox ratio variables. Yellow highlights include correlations between post-treatment value and change with treatment for the same variable.

Supplementary Table S3. Linear modeling of OMI index treatment effect size, change in wH-index with treatment, and other fluorescence lifetime and heterogeneity variables. Heterogeneity and mean FLIM variables defined in Supplementary Figure S17. Adjusted R^2 values quantify the correlation between each dependent variable and a category of independent variables measured. N=401 control/treatment pairs.

	**OMI index Glass's Δ vs. Heterogeneity Variables**	**OMI index Glass's Δ vs. Mean FLIM Variables**	**OMI index Glass's Δ vs. Heterogeneity Variables + Mean FLIM Variables**	**Change in wH-index vs. Heterogeneity Variables**	**Change in wH-index vs. Mean FLIM Variables**	**Change in wH-index vs. Heterogeneity Variables + Mean FLIM Variables**
**Breast Cancer**	0.76	0.92	0.95	0.64	0.03	0.74
**Pancreatic Cancer**	0.34	0.42	0.49	0.47	0.09	0.51
**Both types**	0.45	0.60	0.66	0.45	0.04	0.47
